# MutAid: Sanger and NGS Based Integrated Pipeline for Mutation Identification, Validation and Annotation in Human Molecular Genetics

**DOI:** 10.1371/journal.pone.0147697

**Published:** 2016-02-03

**Authors:** Ram Vinay Pandey, Stephan Pabinger, Albert Kriegner, Andreas Weinhäusel

**Affiliations:** 1 AIT Austrian Institute of Technology, Health and Environment Department, Molecular Diagnostics, Vienna, Austria; 2 Institut für Populationsgenetik, Vetmeduni Vienna, Veterinärplatz 1, A-1210, Vienna, Austria; The University of Hong Kong, HONG KONG

## Abstract

Traditional Sanger sequencing as well as Next-Generation Sequencing have been used for the identification of disease causing mutations in human molecular research. The majority of currently available tools are developed for research and explorative purposes and often do not provide a complete, efficient, one-stop solution. As the focus of currently developed tools is mainly on NGS data analysis, no integrative solution for the analysis of Sanger data is provided and consequently a one-stop solution to analyze reads from both sequencing platforms is not available. We have therefore developed a new pipeline called MutAid to analyze and interpret raw sequencing data produced by Sanger or several NGS sequencing platforms. It performs format conversion, base calling, quality trimming, filtering, read mapping, variant calling, variant annotation and analysis of Sanger and NGS data under a single platform. It is capable of analyzing reads from multiple patients in a single run to create a list of potential disease causing base substitutions as well as insertions and deletions. MutAid has been developed for expert and non-expert users and supports four sequencing platforms including Sanger, Illumina, 454 and Ion Torrent. Furthermore, for NGS data analysis, five read mappers including BWA, TMAP, Bowtie, Bowtie2 and GSNAP and four variant callers including GATK-HaplotypeCaller, SAMTOOLS, Freebayes and VarScan2 pipelines are supported. MutAid is freely available at https://sourceforge.net/projects/mutaid.

## Introduction

Next-Generation Sequencing (NGS) has become a powerful, efficient and cost-effective clinical tool for mutation screening and decoding a number of genetically heterogeneous diseases such as cancer [1]. During the past few years, several clinical studies have already been conducted by applying multi-gene panel tests [2–6], whole exome sequencing [7–9] and whole genome sequencing [10–14]. While NGS technologies have been used to identify variants in several patients in a cost and time effective manner, which typically yields hundreds to several thousands of variants per patients/samples, traditional Sanger sequencing has been used as a complementary method to confirm the NGS-detected variants before making clinical decisions [15].

Currently, the NGS and Sanger data analysis processes, including quality control, mapping, variant identification, validation and clinical annotation, are labor-intensive and cumbersome tasks, which hamper its usage in routine mutation screening. Therefore, a robust and integrated bioinformatics solution, which can be used to analyze several patients in parallel and produces an annotated list of variants from raw sequencing reads, is extremely useful. In addition, it should be possible to run the complete workflow with a single command, and NGS (Illumina, 454 and Ion torrent) as well as Sanger sequencing data analysis should be supported under a single platform.

At present, a few solutions are available for NGS data quality control, mapping, variant calling and variant effect prediction such as ngs_backbone [16], bcbio_nextgen [17], and SIMPLEX [18]. These are widely used in genomic research but do not provide a specific focus for the analysis of clinical samples. Moreover, none of these provide a complete pipeline for mutation screening by Sanger sequencing data analysis or extensive variant annotations. Bcbio_nextgen is a useful tool for short reads mapping, BAM file quality filtering and variant calling but does not provide quality control for raw reads, variant annotation or Sanger sequencing analysis. SIMPLEX is another pipeline for Whole Exome Sequencing (WES) data analysis of Illumina and ABI SOLiD reads, but does not support 454, Ion Torrent or Sanger sequencing data.

In addition to pipeline systems, workflow management tools, such as Galaxy [19], Knime [20] and Chipster [21], are capable of analyzing sequencing data, but do not provide workflows equipped for use in human molecular genetics research and genetic testing settings. Consequently, users do not have an integrated solution to identify variants with NGS data and validate them with Sanger sequencing data.

To address above limitations in data analysis and interpretation for mutation identification, validation and annotation, we present here a new bioinformatics solution, MutAid, a powerful, integrated and easy to use pipeline for Sanger and NGS data analysis. It covers gene panel, exome as well as whole genome sequencing widely used in human molecular genetics. The tool supports one-stop analysis of various sequencing data from raw reads to an annotated variant list. Moreover, aligned reads in BAM/SAM format generated from any sequencing data can be used as direct inputs in MutAid to identify and confirm mutations. MutAid provides a robust and powerful pipeline to analyze NGS sequencing data by supporting five short read mappers including BWA [22], Bowtie [23], Bowtie2 [24], GSNAP [25] and TMAP [26] and four variant callers including GATK-HaplotypeCaller [27], SAMTOOLS [28], Freebayes [29] and VarScan2 [30]. The parallel execution of these mappers and variant callers enable users to quickly select the consensus variants (called by more than one variant caller), which are more reliable than variants only identified by a single tool. For each variant MutAid predicts the variant effect, such as codon change, amino acid change, and frameshift. Furthermore, it cross-references each variant with more than 30 clinically relevant public databases, which contain already reported SNPs and INDELs from previous experimental studies along with associated genomic, proteomic, and clinical information. MutAid can be used to analyze, elucidate and interpret mutational variants from data generated by targeted re-sequencing, gene-panel sequencing, exome, and whole genome sequencing.

## Implementation

The MutAid pipeline uses Python 2.7.9 and implements multiprocessing capabilities to efficiently analyze diagnostic sequencing data produced from Sanger sequencing, Illumina, 454 and Ion Torrent sequencing. It has been designed to specifically consider the Sanger and NGS sequencing chemistries, data format and data quality control requirements. MutAid provides a complete solution for diagnostic sequencing by the seamless integration of traditional Sanger sequencing and high throughput NGS under a single platform. The tool is provided as a fully configured Virtual Machine (VM), which can be used on Windows, Linux and Mac OSX systems with a free Virtual Box installation (https://www.virtualbox.org/). The complete data analysis workflow of MutAid can be started with a simple command and can be customized with many useful parameters. Optionally, the pipeline can be run step-by-step in a modular approach, which allows users to examine the quality of the sequencing data and fine-tune the parameters for specific requirements.

### MutAid pipeline

**Fig 1:**
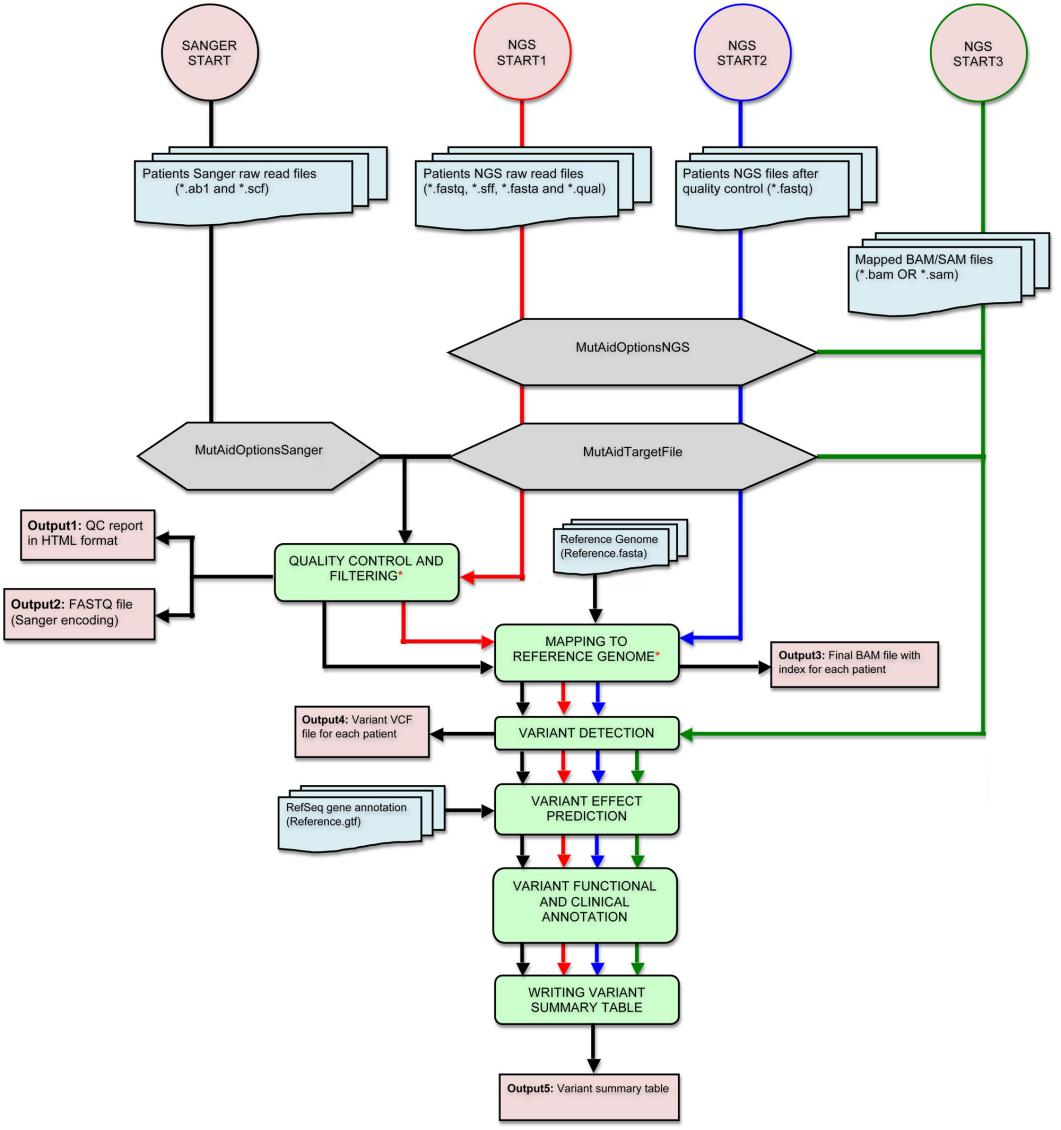
The workflow of MutAid. The MutAid pipeline can be run with a single command. Sanger sequencing data analysis has one start point and the flow of analysis runs from top to the bottom, illustrated by a black arrow. NGS has three starting points: 1) raw reads (red color), 2) high quality FASTQ file (blue color)—in this case first step is skipped and 3) mapped reads in BAM or SAM file format (green color)—in this case step 1 and 2 are skipped. * Step is optional.

The MutAid pipeline (Fig 1) consists of six sequential steps: 1) quality control and filtering, 2) mapping reads to reference genome, 3) variant detection, 4) variant effect prediction, 5) variant annotation and 6) creation of a variant summary table.

As shown in Fig 1, Sanger data analysis has only one start point and always starts with raw read files (AB1, SCF). NGS data analysis has three optional start points in the MutAid pipeline: 1) starts with raw read files (FASTQ, SFF, FASTA-QUAL), 2) starts with FASTQ file with high quality reads, and 3) mapped reads in BAM/SAM file.

#### Step 1. Quality control and filtering

The MutAid quality control algorithm is designed to take care of platform specific quality issues such as different sequencing error rates or variant calling in low quality regions. This step first converts reads from the original format of the sequencer to the FASTQ format with PHRED quality encoding.

The Sanger sequencing read quality trimming and base recalling are done by TraceTuner [31]. Adapters and Primer sequences are trimmed by AlienTrimmer [32], which is a flexible and sensitive sequence trimmer with mismatch tolerance. Finally reads are trimmed based on the specified minimum average base quality. Reads are discarded from further analysis if they do not meet the given minimum base quality or minimum/maximum read length criteria.

After quality trimming and filtering, a detailed QC report is generated using the widely used FASTQC [33] tool. The report contains various useful plots for each FASTQ file to compare quality statistics before and after quality control.

#### Step 2. Mapping reads to reference genome

MutAid maps the sequencing reads to the reference genome followed by post- filtering for minimum mapping quality, proper pair filtering (only for paired end reads), and PCR duplicate removal. By default the long Sanger sequencing and 454 sequencing reads are mapped using the BWA-SW algorithm, which is specially designed for longer reads up to several KB length. For Ion Torrent data, MutAid uses the TMAP mapper, which is tailored to the analysis of Ion Torrent reads. For Illumina data, MutAid uses BWA as the default mapper. MutAid provides three additional mappers including Bowtie, Bowtie2, and GSNAP. Consequently, the user has the option to map a dataset with multiple mappers and compare the resulting variants or select the consensus variants obtained by multiple mappers. The BAM file generated during mapping can be stored or used for re-analysis or co-analysis in other experiments or studies.

#### Step 3. Variant detection

MutAid detects SNVs, insertions and deletions from BAM files using SAMTOOLS for Sanger data and GATK for NGS data as default variant callers. In addition to GATK, MutAid supports three more variant callers for NGS including Freebayes, SAMTOOLS and VarScan2.

After mapping, NGS data is first processed by tools from the GATK bundle including IndelRealigner, BaseRecalibrator and HaplotypeCaller for INDEL realignment, quality recalibration, and variant calling, respectively [34]. Further, the variants are filtered based on read coverage and a minor allele frequency threshold defined in the “*MutAidOption*”file. The resulting realigned BAM files and the VCF files for each patient are generated for further downstream analysis and can be used to visualize the sequences and/or variants in IGV [35].

#### Step 4. Variant effect prediction

Using the variant coordinates, the identified variants are assigned to genomic features (e.g., RefSeq Gene, exon, CDS, intron). In addition, MutAid performs variant effect prediction of SNVs and INDELs. For codon and amino acid changes, MutAid extract the CDS coordinates and reading frame information from gene annotation files. Based on the variant position and reading frame the reference allele is replaced with the variant allele to obtain the variant codon. Once the reference codon and the variant codon have been obtained, the corresponding amino acid from the codon translation table is determined. Both codons and amino acids are reported in the final variant summary table. For INDELs, MutAid defines the frameshift mutation in coding regions by incorporating the inserted and deleted base pair into the actual coding sequence and calculates the new reading frame. Finally, MutAid reports the new reading frame along with the original reading frame in the output table.

To obtain the Gene annotation GTF file, reference genome FASTA file and other annotation files, MutAid uses an additional tool (*prepref*), which downloads all genes and SNP annotations from the UCSC genome browser [36] and prepares them for MutAid analysis.

MutAid calculates the following summary statistics for each mutation: 1) mean and median base quality around each variant in a 10bp flanking region, 2) total sequencing coverage and coverage of A, T, C, G separately before and after quality filtering, 3) p-value before and after multiple testing correction (FDR < = 0.05) calculated using two-tailed fisher’s exact test [37] for each variant in a 2x2 contingency table. A detailed description of Fisher’s exact test is given in S2 Appendix. These summary statistics can be used for sequencing data interpretation and selection of potential candidate mutations.

#### Step 5. Variant cross-referencing

MutAid links each resulting variant with more than 30 relevant databases including gene resources, clinical resources, pathways resources, protein resources, GWAS resources, genome browsers and other databases. The reference information files have been used from the UCSC genome browser and NCBI [38].

For variants, which fall in annotated genes, MutAid first assigns RefSeq gene, transcript, protein IDs as well as Entrez gene ID and a gene symbol. In addition, it constructs for each annotation a link to the databases given in Table 1. For intergenic variants, MutAid constructs links to several genome browsers, such as UCSC and Ensemble, using the variant coordinates as well as to the dbSNP database and the NCBI variation viewer.

**Table 1:** MutAid variant cross-referencing. MutAid constructs direct links to more than 30 publically available databases for each variant in the output summary table. These links are created based on coordinates, and the Entrez gene ID. The table lists, which links will be created for known and novel variants, in exonic and intergenic regions.

Database	Known variants	Novel variants	Intergeneic region variants
**Cross-referencing by coordinate**			
UCSC genome browser	X	X	X
Ensembl genome browser	X	X	X
Decipher	X	X	X
Gwas Central	X	X	X
**Cross-referencing by dbSNP identifier (SNP ID)**			
PolyPhen_2	X		
NCBI dbSNP	X		X
NCBI variation viewer	X		X
**Cross-referencing by Entrez gene ID**			
Entrez Gene	X	X	
ClinVar	X		
dbVar	X		
Genetic Testing Registary (GTR)	X		
WikiGenes	X	X	
BioGPS	X	X	
**Cross-referencing by Entrez gene symbol**			
Cosmic database	X		
GeneTests	X		
GENATLAS	X		
GeneCards	X		
GOPubmed	X		
H_InvDB	X		
**Cross-referencing by RefSeq Transcript ID**			
RefSeq mRNA database	X		
HomoloGene	X		
GEO Profiles	X		
UniGene	X		
Pubmed	X		
**Cross-referencing by RefSeq Protein ID**			
RefSeq Protein database	X		
**Cross-referencing by UniProt Protein ID**			
UniProt	X		
QuickGO	X		
Reactome pathway database	X		
**Cross-refrencing by KEGG pathway ID**			
Kegg pathway database	X		
**Cross-refrencing by OMIM ID**			
OMIM database	X		
**Cross-refrencing byHGNC ID**			
HGNC database	X		

As described in Table 1, cross-referencing by coordinate works for all three types of variants (known, novel and intergenic variants) and cross-referencing by dbSNP identifier (SNP ID) works for known and intergenic variants (except for PolyPhen-2).

#### Step 6. Producing variant summary output

MutAid creates a final output table that contains one line for each variant along with experimental information, patient information, identified mutations and their genomic effects as well as clinical annotations. In addition, it creates links in the output table to the QC report, the mapped BAM file, the variant VCF file and the resulting FASTQ file for each identified mutation. The produced BAM files and VCF files can be visualized in IGV, or directly plugged into other downstream analysis tools and stored or used in other related projects.

### MutAid input

MutAid uses a uniform input and output data model for both Sanger and NGS sequencing data analysis. It can analyze hundreds of sample/patient data in parallel and produces a single variant output file. MutAid requires at least three input files:

#### 1. Target file

A file containing experimental and sequencing file information for each patient (S1 Table).

#### 2. Adapter-Primer file

This input file is optional and is required if primer and adapter sequences need to be trimmed. The tab-separated text file with four columns (S2 Table) is used in the first step (quality control and trimming) of the analysis procedure.

#### 3. MutAidOptions file

This file contains all options for various parts of the pipeline and the path to the installation directory of third party tools including mappers and variant callers. A default MutAidOptions file for Sanger and NGS data analysis is provided separately (S1 and S2 Texts).

#### 4. Sequencing reads

MutAid supports Sanger sequencing reads in AB1 and SCF file format, Illumina reads in FASTQ format, 454 reads in SFF as well as FASTQ-QUAL format and Ion Torrent reads in SFF and FASTQ format. In addition to raw read files, MutAid also supports calling and annotating variants from alignment (BAM/SAM) files. These sequencing data files can be specified in Target file (S1 Table).

### MutAid output

MutAid produces output files in a standardized format for both Sager and NGS, which facilitates post analysis output handling, further downstream analysis and comparison. MutAid returns five output files 1) a variant summary table, 2) a QC report, 3) a VCF file per patient, 4) an indexed BAM file per patient and 5) a Sanger encoded FASTQ file per patient. All these output files will be created in the output directory, which is specified in “*MutAidOption*” file (S1 and S2 Texts).

The variant summary table is the main output, which contains one line for each resulting variant (SNV or INDEL) and links it with a wide range of information as described in Table 2. The other four outputs QC report, FASTQ file, BAM file and VCF files are produced for each sample/patient separately and are linked from each variant in the variant summary table. The detailed QC report is generated in HTML format and includes various useful plots to check the sequencing data. Two QC reports for each patient/sample before and after quality control are generated, which allows direct comparison of quality control result.

**Table 2:** MutAid variant summary output table description. MutAid produces a final variant summary with one line per variant including experimental information, patient information, variant information, variant effects and database cross-references.

Output Info category	Output Info	Output Info Example
**Patient Information**	Patient_Id	P000002
	Family_Id	F01
	Lab_Analysis_Date	2013-10-30_18-42-59
	Seq_Platform	Illumina
	Seq_System	HiSeq2000
	Assay_Id	BRCA_Panel1
**Variant Information**	Var_Id	chr13.GRCh37:g.18258370G>A
	Var_Type	SNV
	Var_Cov	229
	Total_Cov	426
	A	197
	C	0
	G	229
	T	0
	Var_Chr	chr13
	Var_Start	18258369
	Var_End	18258370
	Var_Strand	+
**Variant Genomic Effect**	Var_Gene	NAT2
	Var_RefGene	NM_000015
	Var_Feature	exon_2;CDS_2
	Var_DNA	A>G
	Var_Codon	AAA>AAG
	Var_AA	Lys>Lys
	Frameshift	
**Additional Output files for each Patient**	FASTQC_Report	p000002_1.fq_fastqc_qc_report.html;p000002_2.fq_fastqc_qc_report.html
	Patient_Fastq	p000002_1.fq;p000002_2.fq
	Patient_Bam	p000002.bam
	Patient_Vcf	p000002.vcf
**Reference DB annotation**	Entrez_Gene	URL to Entrez Gene database by Entrez gene ID (10)
	RefSeq_mRNA	URL to RefSeq mRNA nucleotide database by RefSeq Transcript ID (NM_000015)
	RefSeq_Protein	URL to RefSeq Protein database by RefSeq Protein ID (NP_000006)
	HomoloGene	URL to HomoloGene database by RefSeq Transcript ID (NM_000015)
	GEO_Profiles	URL to GEO_Profiles database by RefSeq Transcript ID (NM_000015)
	UniGene	URL to UniGene database by RefSeq Transcript ID (NM_000015)
	Pubmed	URL to PubMed database by RefSeq Transcript ID (NM_000015)
	dbSNP	URL to dbSNP database by SNP identifier (rs1799931)
	ClinVar	URL to ClinVar by Entrez gene ID (10)
	dbVar	URL to dbVar by Entrez gene ID (10)
	NCBI variation viewer	URL to dbSNP database by SNP identifier (rs1799931)
	Cosmic	URL to Cosmic database by Entrez gene symbol (NAT2)
	Gen_Test_Reg	URL to Genetic Testing Registry by Entrez gene ID (10)
	Omim	URL to OMIM by omim ID (612182)
	Hgnc	URL to HGNC by HGNC ID (7646)
	PolyPhen_2	URL to PolyPhen2 database by SNP identifier (rs1799931)
	Decipher	URL to Decipher genome browser by genomic cooridinate (chr8:18258370..18258370)
	Kegg	URL to KEGG pathway by KEGG Pathway ID (hsa03440)
	Kegg_Locus	URL to KEGG pathway Locus by KEGG Pathway ID and Entrez Gene ID(hsa03440 and 675)
	Reactome	URL to Reactome database by UniProt Protein identifier (P51587)
	WikiGenes	URL to WikiGenes by Entrez gene ID (10)
	GeneTes	URL to GeneTes by Entrez gene symbol (NAT2)
	BioGPS	URL to BioGPS by Entrez gene ID (10)
	GENATLAS	URL to GENATLAS by Entrez gene symbol (NAT2)
	GeneCards	URL to GeneCards by Entrez gene symbol (NAT2)
	GOPubmed	URL to GOPubmed by Entrez gene symbol (NAT2)
	H_InvDB	URL to H_InvDB by Entrez gene symbol (NAT2)
	UniProt	URL to UniProt database by UniProt Protein identifier (P51587)
	QuickGO	URL to QuickGO database by UniProt Protein identifier (P51587)
	UCSC	URL to UCSC genome browser by genomic coordinate (chr8:18258370–18258370)
	Ensembl	URL to Ensembl genome browser by genomic coordinate (chr818258370-18258370)
	GWAS_Central	URL to GWAS Central genome browser by genomic cooridinate (chr8:18258370..18258370)
**Interpretation of sequencing data**	Fishers_Exact_Test_pvalue	2.2e-16
	Fishers_Exact_Test_pvalue_FDR_corrected	0,076420371
	dbSnp_Id	rs1801406
	dbSnp_Common	X
	dbSnp_Coding	X
	Gwas_Catalogue	X
	dbSnp_Flagged	X
	dbSnp_Mult	X
	dbSnp_HapMap	X
	dbSnp_Cpg_Island	X
**Mapper & Variant callers**	Mapper_Name	Bwa;Bowtie2;GSNAP
	Variant_Caller	gatk;freebayes;samtools;varscan;hotspot

### Availability, installation and usage

MutAid is an open-source pipeline and freely available for all researchers and clinicians. It is available as a fully configured Virtual Machine accessible at https://sourceforge.net/p/mutaid/wiki/Virtual_Machine/. Moreover, MutAid source code along with pre-compiled required third party tools is available from https://sourceforge.net/projects/mutaid/files/MutAid_v1.0-linux.zip for Linux and from https://sourceforge.net/projects/mutaid/files/MutAid_v1.0-macos.zip for MACOSX operating system. An extensive user manual is available at https://sourceforge.net/projects/mutaid/files/Manual.pdf. Test data sets for Sanger, Illumina, 454 and Ion Torrent are available at https://sourceforge.net/projects/mutaid/files/test_data.zip.

## Results and Discussion

MutAid is an open-source bioinformatics solution, which enables users to analyze both NGS and Sanger sequencing data in human genetic testing. It enables users to rapidly analyze hundreds of patients in parallel by executing just a single command. This pipeline is equipped with various custom parameters and provides proper guidelines for the analysis. All components of MutAid and their inputs are summarized in Fig 1 and all key features are described below.

### An integrated pipeline

Many clinics and hospitals across the globe have been widely using traditional Sanger sequencing for genetic testing, which is still the gold standard even after the advent of NGS technologies. However, it is time consuming and expensive when multiple genes are sequenced. In contrast, NGS platforms are producing billions of short reads at an unprecedented speed in a cost effective manner. Therefore, a single data analysis solution that combines analysis of traditional Sanger sequencing and NGS data could tremendously improve genetic testing in clinical research and diagnostics. With MutAid users can identify the candidate mutations by using NGS sequencing in a time and cost effective manner and validate them with Sanger sequencing. MutAid can be used by expert and non-expert users to get a list of variants from raw sequencing data in a reads-to-variant manner without manual interaction.

### Sanger and NGS integration

MutAid provides an integrated solution for the analysis of Sanger and NGS (Illumina, 454 and Ion Torrent) data analysis under a single platform. It takes a target file as input, which contains sequencing files for all patients/samples along with experimental information for processing. As shown in S1 Fig, Sanger, Illumina, 454 and Ion Torrent have exactly identical target file formats. Thus, the uniform input format enables user to easily prepare a tab delimited input file for different platforms. For both Sanger and NGS, MutAid produces variants in standard output formats, such as VCF and BAM, for each patient/sample, which can be easily visualized. Finally, MutAid creates identical variant summary output tables (Table 2) for both Sanger and NGS data analysis. Hence, identical input, output and visualization models facilitate the direct comparison of variants and confirmation by visual and manual inspection.

### Variant calling validation study

MutAid has been designed to support the parallel execution of analysis runs using several different mapper-variant caller combinations. The results of the different runs can be used to eliminate false positives variants calls from high throughput NGS data analysis. User can use all combinations of mappers and variant callers in a single run and MutAid produces a final comprehensive variant summary output table. In the final summary output table, MutAid reports how many mapper(s) and variant caller(s) have confirmed the resulting variant. In order to demonstrate the capabilities of MutAid, we have designed and executed a variant calling validation study involving three mappers and four variant callers. First we examined the effect of using the same variant caller method but three different mapping tools. In the second part of the study, we used the same mapping result as a base for variant calling with four different variant calling tools. This was executed with four different mapping results.

#### Effect of mapping result on variant calling

**Fig 2:**
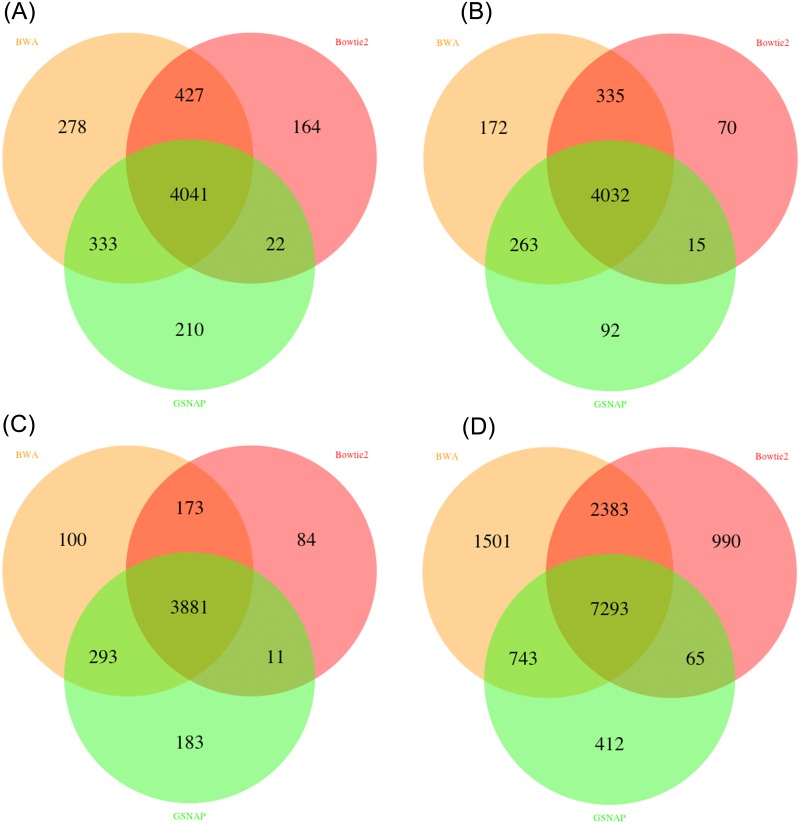
Venn diagrams of called SNVs in MutAid by four variant callers with BWA, Bowtie2 and GSNAP mapping (A) Freebayes (B) GATK-HaplotypeCaller. (C) SAMTOOLS and (D) VarScan2. GATK shows 93.29% overlap between at least two mappers whereas Varscan2 shows least overlap among all four variant callers with 78%. SAMTOOLS and Freebayes show 92.23% and 88%, respectively, agreement with at least two mappers.

**Fig 3:**
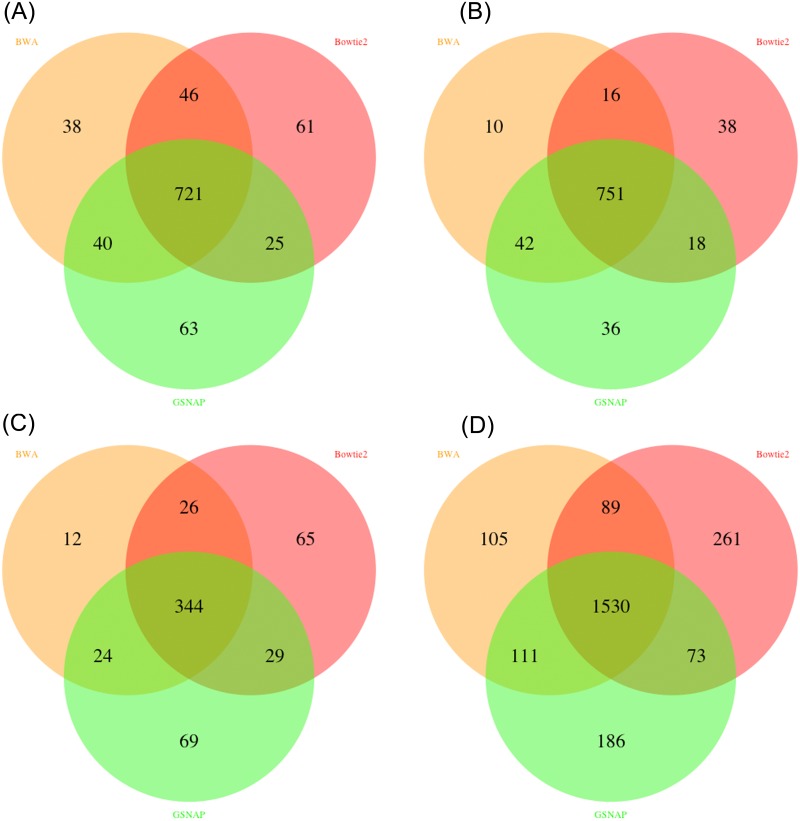
Venn diagrams of called INDELs in MutAid by four variant callers using BWA, Bowtie2 and GSNAP mapping results. (A) Freebayes (B) GATK-HaplotypeCaller. C) SAMTOOLS and (D) VarScan2. GATK shows 90.78% overlap between at least two mappers and SAMTOOLS shows least overlap among all four variant callers with 74.34%. Varscan2 and Freebayes show 76.56% and 83.70%, respectively, agreement with at least two mappers.

First, we compared the called variants after mapping with three mappers; BWA, Bowtie2 and GSNAP. As shown in Figs 2 and 3 the output of the variant callers is highly consistent when using the result of different mapping programs as base for variant calling. For SNV and INDEL identification 76 to 93.29% total variants overlap between at least two mappers. The availability of multiple mappers allows users to evaluate different mapping programs based on their input data and choose the best mapper for further analysis runs.

#### Effect of variant callers on variant calling

In the second part of our validation study we compared the results of different variant calling tools starting with the same mapping result. MutAid supports four variant callers for NGS data analysis. Users can call variants with all four callers simultaneously with a single MutAid command. Furthermore, users can select the variants identified by all four variant callers to get high confidence results. By default SAMTOOLS is used for variant calling of Sanger sequencing data and GATK-HaplotypeCaller for Illumina, 454 and Ion Torrent sequencing.

**Fig 4:**
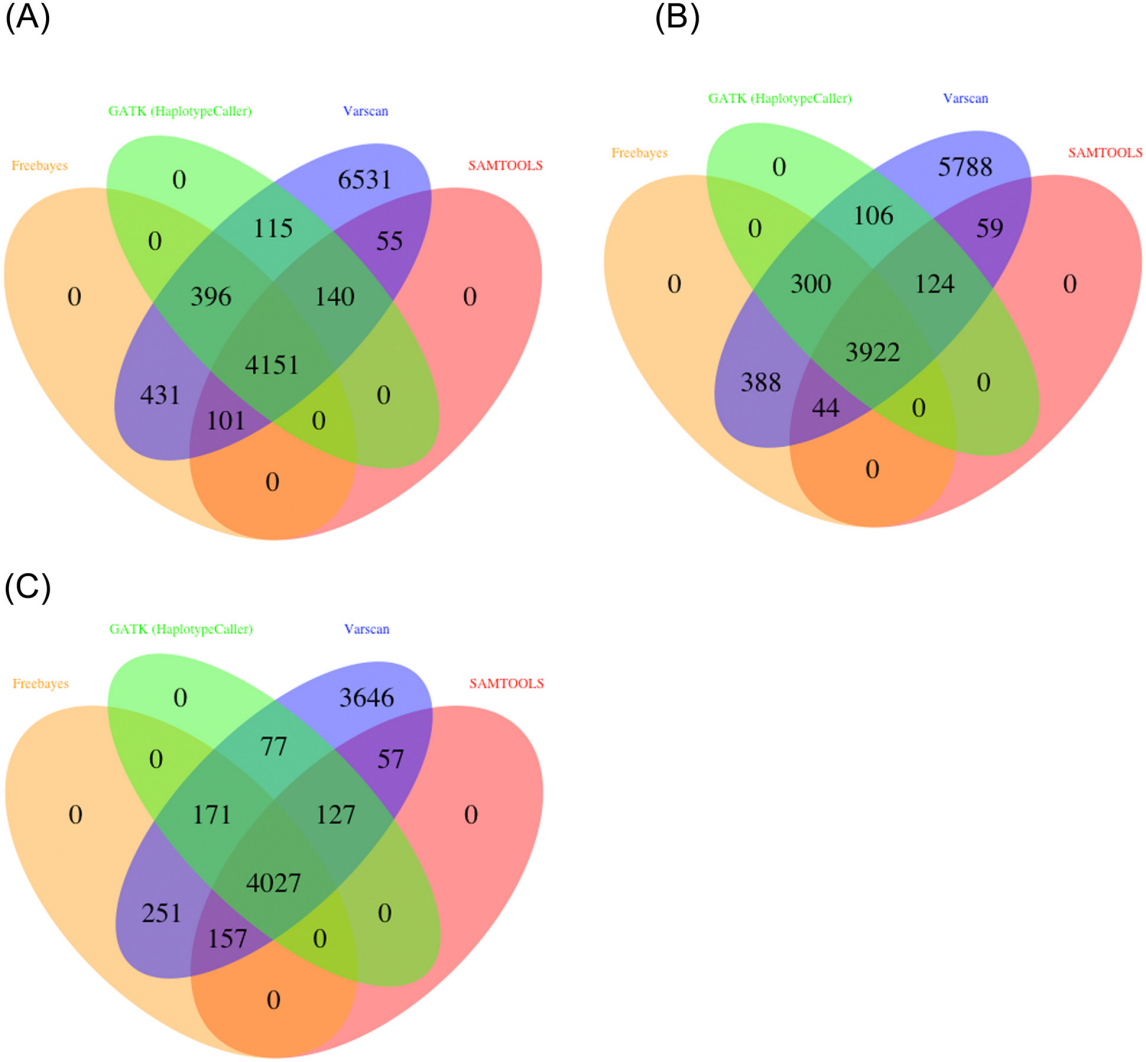
Venn diagrams of called SNV by four variant callers using (A) BWA (B) Bowtie2 (C) GSNAP with same mapper. Result shows that 75% - 84% SNVs are common with at least two out of four variant callers. With all 3 mappers Varscan2 identified novel SNVs from 16% - 24%.

**Fig 5:**
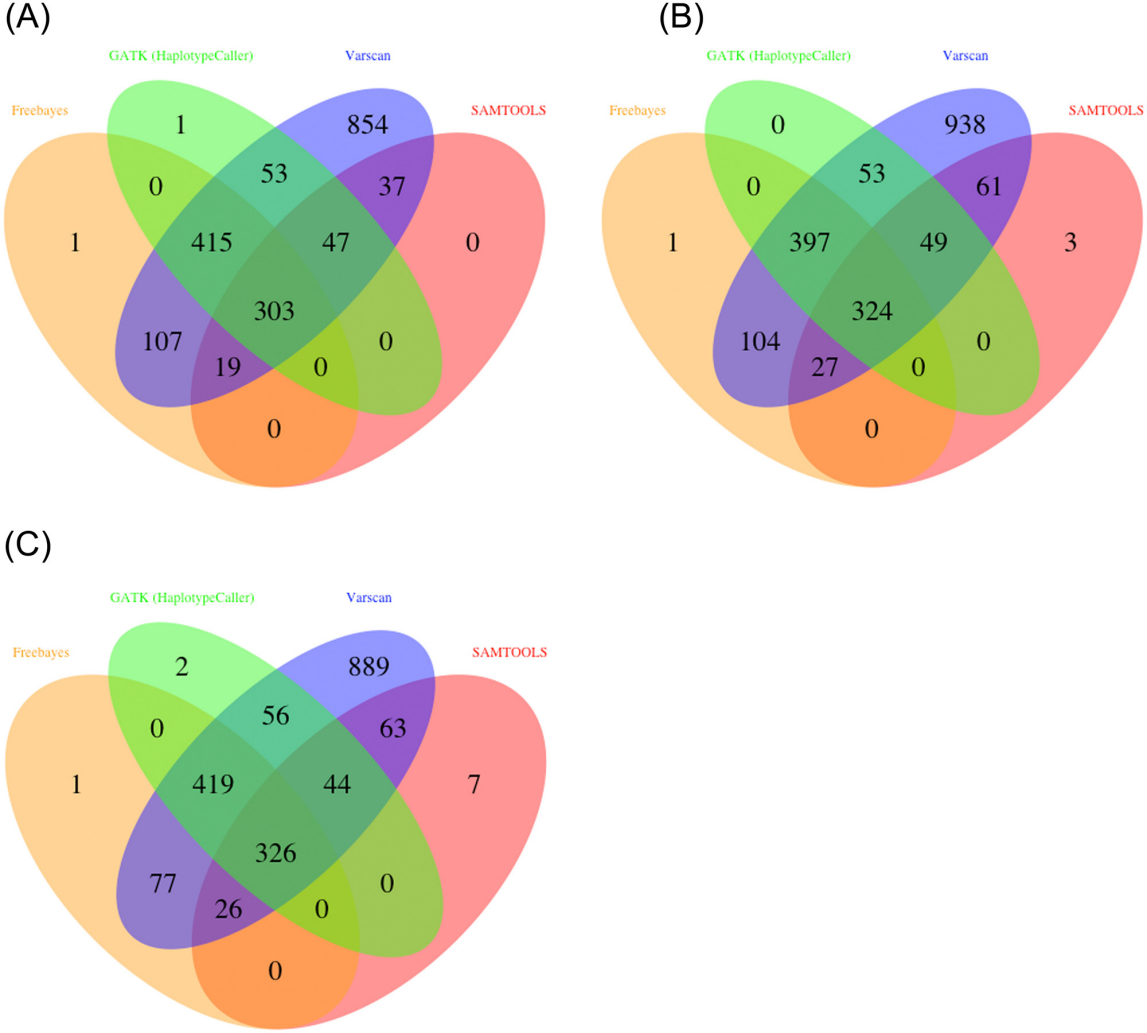
Venn diagrams of called INDEL by four variant callers using (A) BWA (B) Bowtie2 (C) GSNAP with same mapper. Consistent with SNV results more than 78% INDELS are identified by at least two variant callers.

In our validation study, we called variants (SNVs and INDELs) with four variant callers from three mapping files produced by BWA, Bowtie2 and GSNAP and compared the resulting variants. As shown in Fig 4, 75% - 84% SNVs were identified by at least two variant callers. Similarly, more than 78% INDELS have been identified by at least two variant callers (Fig 5).

### Flexibility and scalability

MutAid provides full flexibility to adjust the input parameters for specific requirements. All input files and parameters can be set using the MutAidOption file (S1 and S2 Texts) to run the whole pipeline without user interaction. MutAid provides three start points for NGS data analysis: 1) with original sequencing reads in SFF, FASTA-QUAL or FASTQ format, 2) reads in FASTQ format with PHRED quality encoding after quality control and filtering (starts with mapping), and 3) with mapped reads in BAM/SAM format (starts with variant calling) (Fig 1). MutAid is designed to handle sequencing data ranging from single-gene to whole genome sequencing of several samples/patients. The tool can be used to analyze several patient data in parallel. Furthermore, it employs a multiprocessing concept to use all assigned CPUs to process data efficiently in parallel.

### Quality control report

Employing the correct NGS quality control steps is very crucial to increase the sensitivity and specificity of identified variants. User can check and compare the read quality plots generated by MutAid. This will help to identify the best parameter sets to get high quality reads for mapping and variant calling.

### Data visualization

**Fig 6:**
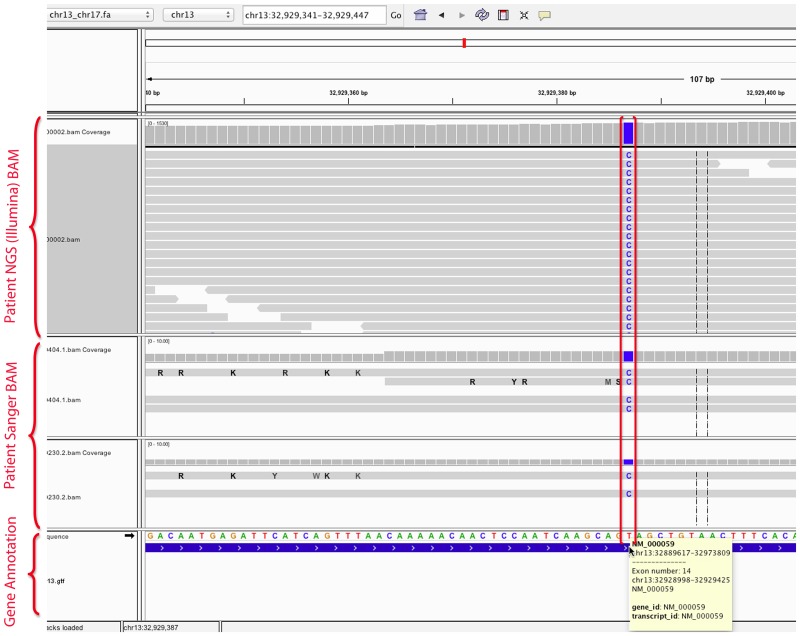
Visualization of SNVs in IGV called by MutAid pipeline with Illumina and Sanger sequencing data analysis. MutAid produces BAM files for NGS and Sanger, which can be loaded into IGV to view and confirm the identified variants. In blue color we can see that SNV (T>C) has been identified by NGS (top panel) and confirmed by Sanger sequencing (middle panel).

Genomic data analysis often requires additional confirmation of the candidate mutation by visual inspection. MutAid provides a direct link for each mutation to visualize it in the UCSC genome browser, the Ensembl genome browser [39] and the GWAS Central browser [40] on their respective web pages. For local visualization in IGV, MutAid produces two output files for each patient 1) a VCF file and 2) a BAM file for both Sanger and NGS. Thus, the user can view and confirm potential pathogenic mutations by visualizing the BAM files in IGV. As shown in Fig 6, a single nucleotide substitution (T>C) in human breast cancer 2 (BRCA2: NM_000059) has been identified with Illumina whole genome sequencing data (top panel) and confirmed by Sanger sequencing reads (middle panel).

### Hotspot mutation annotation

MutAid offers a unique feature to co-analyze and annotate the hotspot mutations along with sequencing data. If a variant overlaps with a known hotspot mutation, it is flagged accordingly in the output table. The user can provide these known hotspot mutations in BED format file (S3 Table).

### Interpretation of sequencing data

To facilitate sequencing data interpretation and selection of potential candidate mutations, MutAid provides the following relevant summary statistics and information for each mutation (columns are described in Table 2):

- Mean and median base quality around each variant in a 10bp flanking window
- Total sequencing coverage and coverage of A, T, C, G separately before and after quality filtering
- Codon and amino acid change for protein coding genes
- Genomic feature assignment (exonic, intronic, UTR, coding region)
- The statistical significance of allele frequency differences is determined with Fisher's exact test. The resulting p-values before and after FDR correction are reported for each variant. The fisher’s exact test is performed with a 2x2 contingency table by using the allele frequencies (ref and alt) of the variant versus the mean allele frequencies of all remaining variants in a sample/patient (S2 Appendix).
- Cross-references and annotations with more than 30 clinically relevant public databases

Based on this readily available information for each identified variant, users will be able to narrow down and confirm potential candidate mutations.

### Interpretation of novel variants

**Fig 7:**
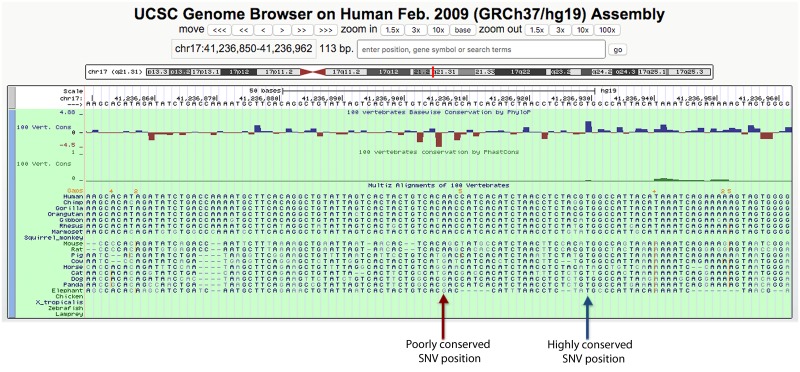
Visualization of conservation track in UCSC genome browser for novel variants. MutAid constructs a direct link to the UCSC genome browser for all variants including novel variants. On top, reference nucleotides are displayed and in the bottom panel (highlighted with green color) the conservation track of several species is displayed. To confirm novel variants, conservation analysis can be performed for each mutation position. A novel mutation might be ignored if a position has poor conservation among the species (pointed by red color arrow). A novel mutation may be further analyzed if the position is highly conserved (pointed by blue color arrow).

Each NGS test may yield several variants that are novel, which poses a great challenge in clinical research, particularly when resources for functional characterization on an individual patient basis are limited [41–42]. MutAid offers useful information for each mutation, which can be readily used to interpret the novel variants. First, MutAid provides summary and quality statistics about each variant, such as base quality, coverage, mapping quality, codon and amino acid change, and frameshift information. Based on this information, users can identify false calls and only variants with high confidence can be selected for further analysis. In addition, MutAid provides a direct link to many genome browsers such as UCSC, Ensembl, Decipher, GWAS Central and NCBI variation viewer for visualization (Table 1), where plenty of genomic and clinical information is available. For instance, users can now consult the UCSC genome browser and analyze the conservation level of the novel variant position (Fig 7). MutAid supports the combined analysis of family members by using the Family ID input field. If a novel mutation is also found in a control sample it may be ignored from further analysis.

### Variant annotation

MutAid provides previously described genetic, gene function and pathway information, which could help to select the clinically relevant variants. As shown in Table 1, MutAid cross-references each variant with more than 30 relevant publically available databases. The resources are described below:

#### Gene resources

In the final output table each variant is cross-referenced to Entrez Gene [43], HomoloGene [44], GEO Profiles [45], UniGene [46] and Pubmed [47]. These resources will be extremely useful for known and novel variant alike. For unknown variants, Pubmed will be particularly useful to search literature and online databases.

#### Clinical resources

MutAid cross-references each variant with the Single Nucleotide Polymorphism Database [48], ClinVar [49], dbVar [50], NCBI variation viewer [51] Cosmic database [52], Genetic Testing Registry [53], Online Mendelian Inheritance in Man (OMIM) [54], HUGO Gene Nomenclature Committee (HGNC) [55], PolyPhen-2 [56] and Decipher database [57].

#### Pathways resources

MutAid links each variant with publically available pathway databases, which provide information about the function of a gene. The linked databases are Kyoto Encyclopedia of Genes and Genomes (KEGG) [58] and Reactome [59]. Before starting the data analysis with MutAid, user need to prepare reference information files (genome FASTA sequence, gene annotation, and variant information) by using the “*prepref*” tool, which is available within MutAid pipeline. *prepref* download RefSeq gene annotation and linked database cross-reference ID of various databases from the UCSC Table browser (http://genome.ucsc.edu/cgi-bin/hgTables). Therefore, after variant identification, the coordinates are assigned to a RefSeq Gene ID, then to KEGG pathway ID (hsa00232) and UniProt Protein ID (P11245). Based on KEGG pathway ID, MutAid creates a direct link to KEGG pathway page and to the Reactome page.

#### Protein resources

MutAid also cross-references protein coding genes with protein databases such as UniProt [60], RefSeq Protein [61] and QuickGO [62].

#### Other databases

Furthermore, MutAid cross-references each variant with other genomic databases such as WikiGenes [63], GeneTes [64], BioGPS [65], GENATLAS [66], Gene Cards [67], GOPubmed [68] and H_InvDB [69].

#### Genome browsers

Each variant is linked to useful genome browsers including UCSC Genome Browser, Ensembl genome browser and GWAS Central browser.

### MutAid validation

To evaluate the MutAid pipeline for mutation screening with NGS data, we used publicly available data (SRP048874) produced from Human Whole Exome Sequencing (WES) of a CEPH/UTAH female individual (HapMap ID: NA12878) (~420 million read pairs, 2x100bp). We downloaded seven sequencing runs from NCBI SRA (SRA ID: SRP048874). After pooling the reads from 7 libraries, data were mapped to the GRCh37/hg19 human reference sequence using the BWA program (0.7.9a) with default parameters. Next, we extracted all reads, which were mapped on chromosome 13 for subsequent analysis.

We ran our MutAid pipeline with ~8 million read pairs. We performed quality control and trimming with minimum read length of 50 base pairs and minimum base quality of 20 and subsequently mapped reads (with passed quality filters) to the chromosome 13 with BWA version 0.7.9a, Bowtie2 version 2.2.3 and GSNAP version gmap-2014-12-16 using their default mapping parameters. We further called variants using MutAid with four variant callers: GATK-HaplotypeCaller version 3.0, SAMTOOLS version 0.1.19, and Freebayes version v0.9.14-14-gb00b735. Next, we filtered variants with minimum read coverage of 20, a minimum variant allele coverage of 4 and variant allele frequency 0.10. The SNVs and INDELs identified by each combination of mappers to variant callers are given in Tables 3 and 4.

**Table 3:** Identified SNVs by MutAid pipeline. SNVs were called using four variant callers (for each mapping result) with a minimum read coverage of 20, minimum variant allele coverage of 4 and a base quality of at least 20. The percentage given in brackets is the fraction of SNVs having an entry in the Single Nucleotide Polymorphism Database (dbSNP) version 137.

	Freebayes (%)	GATK-HaplotypeCaller (%)	SAMTOOLS (%)	Varscan2 (%)
Bowtie2	4654(90.09)	4452(94.77)	4149(97.78)	10731(55.65)
BWA	5079(89.33)	4802(93.77)	4447(97.55)	11920(55.99)
GSNAP	4606(94.03)	4402(96.93)	4368(98.10)	8513(68.80)

**Table 4:** Identified INDELs by MutAid pipeline. INDELs were called using four variant callers (for each mapping result) using the same settings as for SNV calling. The percentage given in brackets is the fraction of INDELs having an entry in dbSNP version 137.

	Freebayes (%)	GATK-HaplotypeCaller (%)	SAMTOOLS (%)	Varscan2 (%)
Bowtie2	853 (39.51)	823 (43.74)	464 (45.91)	1953 (31.29)
BWA	845 (38.46)	819 (41.64)	406 (45.07)	1835 (30.19)
GSNAP	849 (40.40)	847 (42.27)	466 (45.92)	1900 (30.53)

#### 1. Cross-validation of called SNVs with dbSNP database

In order to evaluate our results, we compared the SNVs and INDELs called by MutAid with the Single Nucleotide Polymorphism database (dbSNP v137) [43] to identify the fraction of already known variants reported in dbSNP. Our results show that 55.65% - 98.10% of all identified variants by MutAid with different variant callers were found in dbSNP with a known function (Table 3).

#### 2. Cross-validation of called INDELs with dbSNP

We have also compared the INDELs identified by MutAid with dbSNP and we found that 30.19% - 45.92% INDELs were found in dbSNP. Consistent with SNVs, VarScan2 predicted most de-novo INDELs (~69%), which could not be found in dbSNP (Table 4).

### Comparison with existing tools

MutAid provides a one-stop solution for simultaneous genetic testing of hundreds of patients by using Sanger and NGS sequencing data. MutAid can be run on any Unix system using a single command and has many user-friendly features, which enable non-expert users to identify and validate the disease causing mutations in a time effective manner with great accuracy and reliability. A comparison of the most important features of MutAid to other available tools is given in Table 5. All similar solutions are available for genomic research with no focus on clinical settings. Moreover none of them supports Ion Torrent and Sanger data analysis for disease causing variant detection and confirmation.

**Table 5:** Comparison of various features of MutAid and other tools for NGS and Sanger data analysis.

Features	MutAid v1.0	ngs_backbone v1.4	bcbio-nextgen 0.9.0	SIMPLEX v2.0
Variant annotation	yes	No	no	No
Co-analysis of hotspot mutations	yes	No	no	no
Sanger data analysis	yes	Yes	no	no
Short read mappers	BWA, Bowtie, Bowtie2, TMAP, GSNAP	BWA	BWA, Bowtie2	BWA
Variant callers	GATK-HaplotyperCaller, SAMTOOLS, Freebayes, Varscan2	GATK	GATK, muTect, Freebayes	GATK
Multiple variant callers in one run	yes	No	yes	no
Quality control	yes	yes	no	yes
Sequencing data supported	targeted sequencing, exome sequencing and whole genome sequencing	transcriptome sequencing	exome sequencing, genome sequencing and transcriptome sequencing	exome sequencing
Several data analysis in single run	yes	no	yes	no
Virtual Machine	yes	no	yes	yes
Installation required	no	yes	no	no
Supported sequencing platforms	Sanger, Illumina, 454, Ion torrent	454, Illumina, ABI SOLiD	Illumina, 454	Illumina, ABI SOLiD
Parallel processing	yes	no	yes	yes
Multiple dataset parallel analysis	yes	no	no	yes
Dependency for installation	no	yes	no	yes
Graphical QC report	yes	no	no	yes

## Conclusions

MutAid is a new robust, user-friendly, and integrated bioinformatics pipeline to analyze NGS and Sanger sequencing data with a single command. The pipeline takes raw reads as inputs and outputs a list of annotated variants with great accuracy for hundreds of patients. It is a useful tool to analyze up to thousands of Sanger sequencing trace files automatically in batch mode and produces a comprehensive single output table of identified substitution, insertion and deletion.

In clinical research and diagnostics, NGS and traditional Sanger sequencing are being used as complementary techniques for mutation screening. Consequently, an integrated bioinformatics solution, which offers NGS and Sanger data analysis under a single platform, provides great potential. We believe that MutAid will be very useful in human genetic testing and diagnostic mutation screening when research and diagnostics are coalescent.

Despite the different nature of Sanger and NGS input files, MutAid provides a common input and output interface to facilitate streamlined use and evaluation of detected variants. With the integration of four variant callers, MutAid provides a powerful approach to select the consensus variants among all variant callers, thus lowering the false positive rate.

The source code along with its documentation is freely available under the AGPL license and can be obtained from https://sourceforge.net/projects/mutaid.

## Supporting Information

S1 Table Target file (mandatory input file) to run MutAid. (TXT)

S2 Table Adapter-Primer file (optional input file) to perform adapters and PCR primers trimming. (TXT)

S3 Table Hotspot mutation file in BED format to co-analyze and annotate with MutAid pipeline. (TXT)

S1 Text MutAidOptions_Sanger file to specify all input files and parameters for Sanger sequencing data analysis. (TXT)

S2 Text MutAidOptions_NGS file to specify all input files and parameters for NGS data analysis. (TXT)

S1 Appendix MutAid user manual. An extensive guide for user to perform Sanger and NGS data analysis with MutAid
(PDF)

S2 Appendix MutAid supplementary method (PDF)

S1 Fig Shows the uniform Target file implementation for Sanger and NGS in MutAid. (TIF)

## Abbreviations

NGS: Next Generation Sequencing
WGS: Whole Genome Sequencing
WES: Whole Exome Sequencing
GTF: Gene Transfer Format
SFF: Standard Flowgram Format
dbSNP: Single Nucleotide Polymorphism Database
SNV: Single Nucleotide Variant
INDEL: Insertion-Deletion
VCF: Variant Call Format
BED: Browser Extensible Data
BAM: Binary Alignment Map
SAM: Sequence Alignment Map
QC: Quality Control
IGV: Integrative Genomics Viewer
NCBI: National Center for Biotechnology Information
SRA: Short Read Archive
