# Supplementary material for: MutAid: Sanger and NGS Based Integrated Pipeline for Mutation Identification, Validation and Annotation in Human Molecular Genetics
Source: PLoS One. 2016 Feb 3;11(2):e0147697. doi: 10.1371/journal.pone.0147697 (PMC4739551; doi:10.1371/journal.pone.0147697)
Supplement: S1 Appendix — An extensive guide for user to perform Sanger and NGS data analysis with MutAid (PDF) [file pone.0147697.s006.pdf]

MutAid User Guide, Version 1.0

Health & Environment Department
AIT Austrian Institute of Technology GmbH
Vienna 1190, Austria
December 21, 2015

TABLE OF CONTENTS

1. What is MutAid? ………………………………………………………………… 3

2. System requirements …………………………………………………………... 4

3. Software/tools required ……………………………………………………….. 4

4. How to obtain MutAid? ……………………………………………………………… 6

    4.1 Download MutAid Virtual Machine ………………………………….. 6

        4.1.1 How to use MutAid Virtual Machine? …………………….. 6

    4.2 Download MutAid source code ………………………….………… 7

    4.3 Run MutAid with test inputs ………………………………………….. 8

5. MutAid inputs requirements …………………………………………….…... 8

6. How to use MutAid? ……………………………………………………….…... 9

    6.1 Prepare reference input files ………………………………………… 9

    6.2 Sanger data analysis …………………………………………….…... 10

    6.3 NGS data analysis ……………………………………………….…... 14

7. MutAid outputs description ………………………………………………….... 19

8. Contact information …………………………………………………………… 20

1. What is MutAid?

MutAid is an integrated pipeline for mutation screening in clinical research. It can analyze Sanger sequencing and NGS data from raw reads to list of annotated mutation list with little or no manual work. The important features of MutAid are described below:

1. MutAid supports three major NGS platforms including Illumina, Roche and Ion torrents.
2. MutAid supports Sanger sequencing data analysis from trace file to list of variants with extensive clinical annotation.
3. MutAid can be used to analyze the sequencing data generated from single-gene-panel, multigene-panel, exome-seq, and genome-seq experiments
4. MutAid has uniform input and output model for Sanger and NGS data analysis.
5. MutAid supports five mappers including BWA, Bowtie, Bowtie2, TMAP and GSNAP to cover a wide range of NGS experiments.
6. MutAid supports four variant callers including GATK-HaplotypeCaller, Freebayes, SAMTOOLS and Varscan2 to identify the SNV and INDEL from NGS sequencing data.
7. MutAid can be used to analyze several patients/samples in a single run simultaneously.
8. To reduce the false positive and increase the sensitivity and specificity user can select the consensus variants from four variant callers and five mappers output.
9. Step 1 (Quality control and filtering) can be skipped if user has sequencing data in Sanger encoded FASTQ file format.

10. Pipeline can be started from Step 3 onwards if sequencing data is readily available in SAM/BAM format. This feature enables user to analyze NGS data produced from any sequencing platforms.

2. System requirements

Operating system

Linux

Mac OSX 10.6 or later

Softwares

Python 2.7.9

Biopython 1.60 or higher

Perl 5.10 or higher

Java 1.7 or higher

R version 2.15.0 or higher

3. Softwares/tools required

Quality control and trimming

*AlienTrimmer [ftp://ftp.pasteur.fr/pub/gensoft/projects/AlienTrimmer/]

*TraceTuner [https://sourceforge.net/projects/tracetuner/]

*FASTQC [http://www.bioinformatics.babraham.ac.uk/projects/fastqc/]

Short read mapping

*BWA [http://bio-bwa.sourceforge.net/]

Bowtie [http://bowtie-bio.sourceforge.net/index.shtml]

Bowtie2 [http://bowtie-bio.sourceforge.net/bowtie2/index.shtml]

TMAP [https://github.com/iontorrent/TS/tree/master/Analysis/TMAP]

GSNAP [http://research-pub.gene.com/gmap/]

Variant detection

GATK [https://www.broadinstitute.org/gatk/]

*SAMTOOLS [https://sourceforge.net/projects/samtools/]

BCFTOOLS

[https://sourceforge.net/projects/samtools/files/samtools/1.2/bcftools-

1.2.tar.bz2]

Freebayes [https://github.com/ekg/freebayes]

Varscan2 [http://varscan.sourceforge.net/]

*PICARD [http://broadinstitute.github.io/picard/]

Other tools

*BedTools [https://github.com/arq5x/bedtools2]

genePredToGtf

[http://hgdownload.soe.ucsc.edu/admin/exe/linux.x86_64/genePredToGtf]

Note: *denotes the Sanger analysis software/tools requirement

4. How to obtain MutAid?

We provide source code in two version of MutAid for Linux and MAC-OSX computers. For Windows PC users we provide a fully configured Virtual Machine (VM can be used on any operating system). Along with source code and virtual machine we provide test data and extensive user manual for step-by-step get and run MutAid for expert and non-expert users.

4.1 Download MutAid Virtual Machine

For all users we provide a fully configured Virtual Machine (VM), which does not require any installation and configuration and works on any operating system including Windows, Linux and Mac osx. The VM can be obtained from https://sourceforge.net/p/mutaid/wiki/Virtual_Machine/

4.1.1 How to use MutAid Virtual Machine?

Step1: Download and install Virtual Box from https://www.virtualbox.org/

Step2: Download MutAid Virtual machine from https://sourceforge.net/p/mutaid/wiki/Virtual_Machine

Step3: Import MutAid Virtual Machine file into Virtual Box

Step4: Login into MutAid Virtual machine with username and password = **testuser**

Step5: Run the MutAid with the test data sets

Step6: For new data analysis with MutAid, prepare the Target file and MutAidOption file to run the MutAid pipeline.

4.2 Download MutAid source code

If user wants to use MutAid on own system then user can download the latest version of MutAid source code (1) for Linux computers from https://sourceforge.net/projects/mutaid/files/MutAid_v1.0-linux.zip and 2) for Macintosh OSX computers form https://sourceforge.net/projects/mutaid/files/MutAid_v1.0-macos.zip. Move the file to an appropriate directory and run the following command to uncompress the file:

unzip MutAid_v1.0-linux.zip

Note that after uncompressing the **.zip** file, a new folder will be created named ***MutAid_v1.0***. This directory contains the following files and folders. Files are denoted in blue and sub folders are denoted in red colors:

< MutAid_v1.0>
|
- < mutaid>
- <MutAidOptions_Sanger>
- <MutAidOptions_NGS>
- <prepref>
- <bin>
- <executables>
  |
  - <AlienTrimmer>
  - <bcftools>
  - <bedtools>
  - <bowtie>
  - <bowtie2>
  - <bwa>
  - <FastQC>
  - <freebayes>
  - <genePredToGtf>
  - <GenomeAnalysisTK>
  - <gmap-gsnap>
  - <picard>
  - <samtools>
  - <TMAP>
  - <TraceTuner>
  - <VarScan>

4.3 Run MutAid with test input

To validate the installation of the MutAid pipeline, it can be run with a small test data set. The test data set and the corresponding MutAid configuration files for Sanger and NGS can be obtained from https://sourceforge.net/projects/mutaid/files/test_data.zip and download in MutAid_v1.0 folder/directory and run the following command to uncompress the file:

unzip test_data.zip

Note that after uncompressing the **.zip** file, a new folder will be created named ***test_data*** in MutAid_v1.0 folder. And then run following two commands to run Sanger data analysis and NGS data analysis.

Sanger analysis:

cd ~/MutAid_v1.0

./mutaid --option_file MutAidOptions_Sanger

NGS analysis:

cd ~/MutAid_v1.0

./mutaid --option_file MutAidOptions_NGS

To get help on how to run MutAid and required parameters enter:

./mutaid –help

5. MutAid inputs requirements

MutAid pipeline consists of six sequential steps, which can be run by a single command. All input parameters can be specified in the **MutAidOptions file**. These parameters are then used to run the whole pipeline. MutAid provides two different input options file for Sanger and NGS:

MutAidOptions_Sanger: This configuration file can be used for Sanger sequencing data analysis for mutation screening. We have already given default value for required parameters to run the whole Sanger sequencing

analysis from raw reads to variation list.

MutAidOptions_NGS: This configuration file can be used for NGS sequencing data analysis for mutation screening. We have already given default value for required parameters to run the whole NGS sequencing analysis from raw reads to variation list.

MutAid can be run with the following command, which should be run under the folder/directory *MutAid_v1.0* directory:

MutAid_v1.0/*mutaid --option_file <path to MutAidOptions file>*

However, before running MutAid with your own dataset all parameters have to be specified in the appropriate MutAid Options files (*MutAidOptions_Sanger* OR *MutAidOptions_NGS*).

6. How to use MutAid?

Before starting the data analysis with MutAid, user need to prepare reference information files (genome FASTA sequence, gene annotation, and variant information) by using the "*prepref*" tool, which is available within MutAid pipeline. prepref downloads RefSeq gene annotation and linked database cross-reference ID of various databases from the UCSC Table browser (http://genome.ucsc.edu/cgi-bin/hgTables). The reference files need to be prepared only once.

Thus following three steps are required to use MutAid with your Sanger or NGS data analysis.

6.1 Prepare reference input files

MutAid uses RefSeq reference genome FASTA file, RefSeq gene annotation file and other SNP and INDEL annotation files from UCSC table browser. We

provide a tool in MutAid to do it automatically by running the following commands.

Go to MutAid source directoy with following command

```
cd ~/MutAid_v1.0
```

Run following command to prepare reference files by giving two parameters:

(1) Genome assembly hg19 or hg38

(2) dbSNP build number 137 or 141 or 142

```
./prepref --genome_assembly hg19 --dbSNP_version 142
```

After successful completion of this tool it creates a ***ref_input*** folder in MutAid_v1.0 folder. The path of reference folder is ***MutAid_v1.0/ref_input***

In this step following reference files will be downloaded and prepared for MutAid analysis. Note that this command needs to be run only in the beginning. Afterwards MutAid can use these files for all Sanger and NGS data analysis.

1. Genome FASTA file
2. RefSeq Genome Gene annotation GTF
3. Genome Gene database cross references
4. Genome SNP and INDEL
5. GATK Bundle (only used for NGS data analysis)

6.2 Sanger data analysis

Step1: Prepare Input files:

1. Prepare Target file

For each analysis user need to prepare a target file in a predefined format. It is a *tab-separated* text file, which contains 10 columns. As shown in figure 1, one row for each sequencing file in target file. Target file is a mandatory input, which must be provided. The target file can be given in the MutAidOptions file with the input name **Target_File="sanger_target_file.txt"**

Patient_Id	Family_Id	Assay_Id	Lab_Analysis_Date	Platform	Seq_System	Read_Type	File_Path	File_Format	Barcode
OO230.2	OO230	ASSanger1	2014-01-27_15-55-12	Sanger	ABI Seq	Single-End	test_data/sanger/ab1/OO230_2/1866_001_1_OO230_2_BR1EX9_F_O01.ab1	ab1	
OO230.2	OO230	ASSanger1	2014-01-27_15-55-13	Sanger	ABI Seq	Single-End	test_data/sanger/ab1/OO230_2/1866_002_1_OO230_2_BR1EX8_F_M01.ab1	ab1	
OO230.2	OO230	ASSanger1	2014-01-27_15-55-13	Sanger	ABI Seq	Single-End	test_data/sanger/ab1/OO230_2/1866_003_1_OO230_2_BR1EX7_2_F_K01.ab1	ab1	
OO230.2	OO230	ASSanger1	2014-01-27_15-55-13	Sanger	ABI Seq	Single-End	test_data/sanger/ab1/OO230_2/1866_004_1_OO230_2_BR1EX7_1_F_I01.ab1	ab1	
OO230.2	OO230	ASSanger1	2014-01-27_15-55-13	Sanger	ABI Seq	Single-End	test_data/sanger/ab1/OO230_2/1866_005_1_OO230_2_BR1EX6_F_G01.ab1	ab1	
OO230.2	OO230	ASSanger1	2014-01-27_15-55-13	Sanger	ABI Seq	Single-End	test_data/sanger/ab1/OO230_2/1866_006_1_OO230_2_BR1EX5_F_E01.ab1	ab1	
OO230.2	OO230	ASSanger1	2014-01-27_15-55-13	Sanger	ABI Seq	Single-End	test_data/sanger/ab1/OO230_2/1866_007_1_OO230_2_BR1EX3_F_C01.ab1	ab1	
OO230.2	OO230	ASSanger1	2014-01-27_15-55-13	Sanger	ABI Seq	Single-End	test_data/sanger/ab1/OO230_2/1866_008_1_OO230_2_BR1EX2_F_A01.ab1	ab1	
OO230.2	OO230	ASSanger1	2014-01-27_15-55-01	Sanger	ABI Seq	Single-End	test_data/sanger/ab1/OO230_2/1866_009_1_OO230_2_BR1EX14_F_O05.ab1	ab1	
OO230.2	OO230	ASSanger1	2014-01-27_15-55-01	Sanger	ABI Seq	Single-End	test_data/sanger/ab1/OO230_2/1866_010_1_OO230_2_BR1EX13_F_M05.ab1	ab1	
OO230.2	OO230	ASSanger1	2014-01-27_15-55-01	Sanger	ABI Seq	Single-End	test_data/sanger/ab1/OO230_2/1866_011_1_OO230_2_BR1EX12_F_K05.ab1	ab1	
OO230.2	OO230	ASSanger1	2014-01-27_15-55-01	Sanger	ABI Seq	Single-End	test_data/sanger/ab1/OO230_2/1866_012_1_OO230_2_BR1EX11l_F_I05.ab1	ab1	
OO230.2	OO230	ASSanger1	2014-01-27_15-55-01	Sanger	ABI Seq	Single-End	test_data/sanger/ab1/OO230_2/1866_013_1_OO230_2_BR1EX11k_F_G05.ab1	ab1	
OO230.2	OO230	ASSanger1	2014-01-27_15-55-02	Sanger	ABI Seq	Single-End	test_data/sanger/ab1/OO230_2/1866_014_1_OO230_2_BR1EX11j_F_E05.ab1	ab1	
OO230.2	OO230	ASSanger1	2014-01-27_15-55-02	Sanger	ABI Seq	Single-End	test_data/sanger/ab1/OO230_2/1866_015_1_OO230_2_BR1EX11i_F_C05.ab1	ab1	
OO230.2	OO230	ASSanger1	2014-01-27_15-55-02	Sanger	ABI Seq	Single-End	test_data/sanger/ab1/OO230_2/1866_016_1_OO230_2_BR1EX11h_F_A05.ab1	ab1	
OO230.2	OO230	ASSanger1	2014-01-27_15-55-02	Sanger	ABI Seq	Single-End	test_data/sanger/ab1/OO230_2/1866_017_1_OO230_2_BR2EX6_7_F_O09.ab1	ab1	
OO230.2	OO230	ASSanger1	2014-01-27_15-55-02	Sanger	ABI Seq	Single-End	test_data/sanger/ab1/OO230_2/1866_018_1_OO230_2_BR2EX5_6_F_M09.ab1	ab1	
OO230.2	OO230	ASSanger1	2014-01-27_15-55-02	Sanger	ABI Seq	Single-End	test_data/sanger/ab1/OO230_2/1866_019_1_OO230_2_BR2EX4_F_K09.ab1	ab1	
OO230.2	OO230	ASSanger1	2014-01-27_15-55-02	Sanger	ABI Seq	Single-End	test_data/sanger/ab1/OO230_2/1866_020_1_OO230_2_BR2EX3_2_F_I09.ab1	ab1	
OO230.2	OO230	ASSanger1	2014-01-27_15-55-03	Sanger	ABI Seq	Single-End	test_data/sanger/ab1/OO230_2/1866_021_1_OO230_2_BR2EX3_1_F_G09.ab1	ab1	
OO230.2	OO230	ASSanger1	2014-01-27_15-55-03	Sanger	ABI Seq	Single-End	test_data/sanger/ab1/OO230_2/1866_022_1_OO230_2_BR2EX2_F_E09.ab1	ab1	
OO230.2	OO230	ASSanger1	2014-01-27_15-55-03	Sanger	ABI Seq	Single-End	test_data/sanger/ab1/OO230_2/1866_023_1_OO230_2_BR1EX24_F_C09.ab1	ab1	
OO230.2	OO230	ASSanger1	2014-01-27_15-55-03	Sanger	ABI Seq	Single-End	test_data/sanger/ab1/OO230_2/1866_024_1_OO230_2_BR1EX23_F_A09.ab1	ab1	

Figure 1: Shows Target file for Sanger data analysis

2. Prepare MutAidOptions_Sanger file

MutAid requires an input configuration file, which can be prepared once by customizing parameters as per the requirement, and the whole pipeline will be run without further user interaction.

(1) Set the **<u>Global input parameters</u>** as shown in Figure 2 below

```
##########################################################################################################################
#
###             Global input parameters
#
##########################################################################################################################

# Give the path to the fasta file of the genomic reference. The reference fasta should be obtained from UCSC genome browser
REFERENCEE_FASTA = ref_input/hg19_fa/chrAll.fa

# Give the gene annotation file of your genomic reference in GTF format. The reference GTF should be obtained from UCSC genome browser
REFERENCE_GTF_FILE = ref_input/hg19_gtf/hg19.gtf

### Give the number of processors that should be used in MutAid pipeline.
THREAD = 10

### Reference genome information from UCSC genome browser
Genome_Assembly = hg19
Refseq_Genome_Build = GRCh37


### dbSNP version from UCSC genome browser
dbSNP_Version = 142
```

Figure 2: Global input parameters of the MutAid pipeline.

(2) Set the **<u>Output file and directory location</u>** as shown in Figure 3 below

```
##########################################################################################################################
#
#                                     Output file and directory location
#
##########################################################################################################################

### Provide the output directory
Output_Dir = "test_output/sanger_output_dir"

### Provide the final output file name
Output_File = "test_output/sanger_output_file"
```

Figure 3: Set Output director and output file name and path

(3) Set the **<u>Quality control and filtering</u>** as shown in Figure 4 below

```
#####################################################################################################################
#
###             Step1: Quality control and filtering
#
#####################################################################################################################

### Minimum average base quality for trimming the low quality 5' and 3' end of reads
Minimum_Base_Quality = 20

### Minimum read length to discard the read for further analysis
Minimum_Read_Length = 50

### Maximum read length to discard the read for further analysis
Maximum_Read_Length = 1000

### Sanger data quality control and trimming parameters for TTUNER
Sanger_Trim_Window_Size = 10
Sanger_Trim_Base_Quality = 20
```

Figure 4: Set Quality control and filtering parameters

(4) Set the **<u>Mapping to reference genome</u>** parameters as shown in Figure 5 below For Sanger data analysis default mapper is set as BWA mapper and Minimum mapping quality threshold 20.

```
#####################################################################################################################
#
###             Step2: Mapping to reference genome
#
#####################################################################################################################

### Mapper name only one mapper at a time allowed. Example Mapper_Name = bwa
Mapper_Name = bwa

### Provide the Mapper_Parameters
Mapper_Parameters = ""
BWA_Sampe_Parameters = ""

### Filtering Mapped reads from BAM/SAM file
Minimum_Mapping_Quality = 20
```

Figure 5: Set Mapping parameters

(5) Set the **<u>Variant detection</u>** parameters as shown in Figure 6 below

```
#####################################################################################################################
#
###             Step3: Variant detection
#
#####################################################################################################################

### Variant callers name.
Variant_Caller = samtools


### SNV and INDEL calling parameters
### Minimum read coverage to call variant
Minimum_Coverage = 1

### Maximum read coverage to call variant. This is useful to discard the repetative region.
Maximum_Coverage = 1000

### Minimum Variant allele read count to consider as variant position
Minimum_Count = 1

### Minimum allele frequency of Variant allele to consider as variant position
Minor_Allele_Frequency = 0.01


### Provide the Hotspot mutation in bed format which will be co-analyzed.  (optional)
Hotspot_Bed_File = ""
```

Figure 6: Set Variant detection and filtering parameters

(6) Set the **<u>Variant functional annotation</u>** as shown in Figure 7 below

```
##############################################################################################################
#
###              Step5: Variant functional annotation
#
##############################################################################################################

## Various SNPs information file from UCSC Table browser
## Provide the full path to all these files.

UCSC_All_SNPs = ref_input/hg19_ucsc_dbxref/chrAll_snpAll.txt

UCSC_Common_SNPs = ref_input/hg19_ucsc_dbxref/chrAll_snpCommon.txt

UCSC_Flagged_SNPs = ref_input/hg19_ucsc_dbxref/chrAll_FlaggedSNPs.txt

UCSC_HapMap_SNPs = ref_input/hg19_ucsc_dbxref/chrAll_HapMapSNPs.txt

UCSC_Multi_SNPs = ref_input/hg19_ucsc_dbxref/chrAll_MultSNPs.txt

UCSC_COSMIC_SNPs = ref_input/hg19_ucsc_dbxref/chrAll_cosmic.txt

UCSC_GWAS_Catalog_SNPs = ref_input/hg19_ucsc_dbxref/chrAll_gwasCatalog.txt

UCSC_CpG_Islands_SNPs = ref_input/hg19_ucsc_dbxref/chrAll_cpgIslandExt.txt

UCSC_Coding_DbSnp = ref_input/hg19_ucsc_dbxref/chrAll_CodingDbSnp.txt

UCSC_OrthoPt3Pa2Rm2_SNPs = ref_input/hg19_ucsc_dbxref/chrAll_OrthoPt3Pa2Rm2.txt

UCSC_RefseqGene_Info = ref_input/hg19_ucsc_dbxref/chrAll_RefseqGene_Info.txt
```

Figure 7: Set the file path of these UCSC reference files

(7) Set the **<u>Third party software/tool executable path</u>** as shown in Figure 8 below

```
##############################################################################################################
#
# Third party software/tool executables
# Provide the full path to the third party executables to run the MutAid pipeline.
#
#
##############################################################################################################


### Get samtools to manipulate SAM and BAM files from http://samtools.sourceforge.net/
# Provide the full path of the executables relative to the <MutAid_1.0>
###### SAMTOOLS path
SAMTOOLS = executables/samtools-1.2/samtools
BCFTOOLS = executables/bcftools-1.2/bcftools


### Define the full path of the Mappers
BWA = executables/bwa-0.7.9a/bwa


###### FASTQC executables
FASTQC = executables/FastQC/fastqc


###### Sanger ab1 and scf file QC tool
TTUNER = executables/tracetuner_3.0.6beta/rel/Linux_64/ttuner
AlienTrimmer = executables/AlienTrimmer_0.4.0/src/AlienTrimmer.jar


#### PICARD JAR file from http://picard.sourceforge.net/index.shtml
###### 3 PICARD jar files are required for MutAid pipeline
PICARD = executables/picard-tools-1.130/picard.jar
BuildBamIndex = executables/picard-tools-1.115/picard-tools-1.115/BuildBamIndex.jar
MergeSamFiles = executables/picard-tools-1.115/picard-tools-1.115/MergeSamFiles.jar
SortSam = executables/picard-tools-1.115/picard-tools-1.115/SortSam.jar


### Get intersectBed executables from a collection of useful utilities called bedtools-
### from http://code.google.com/p/bedtools/
###### 3 bedtools
intersectBed = executables/bedtools2/bin/intersectBed
bedtools = executables/bedtools2/bin/bedtools
bamToBed = executables/bedtools2/bin/bamToBed
```

Figure 8: Specifications of full paths of external software/tools used in MutAid.

Step2: Run MutAid pipeline:

After preparing the Target File and MutAidOptions file have been prepared and customized then MutAid pipeline can be run with following command line

MutAid_v1.0/mutaid –option_file MutAidOptions_Sanger

6.3 NGS data analysis

Step1: Prepare Input files:

1. Prepare Target file

For each analysis user need to prepare a target file in a predefined format. It is a *tab-separated* text file, which contains 10 columns. As shown in below figure 9, one row for each sequencing file in target files. Target file is a mandatory input, which must be provided. The target file can be given in the MutAidOptions file with the input name **Target_File="ngs_target_file.txt"**

(a)

Patient_Id	Family_Id	Assay_Id	Lab_Analysis_Date	Platform	Seq_System	Read_Type	File_Path	File_Format	Barcode
OO230.2	OO230	ASSanger1	2014-01-27_15-55-12	Sanger	ABI Seq	Single-End	test_data/sanger/ab1/OO230_2/1866_001_1_OO230_2_BR1EX9_F_O01.ab1	ab1	
OO230.2	OO230	ASSanger1	2014-01-27_15-55-13	Sanger	ABI Seq	Single-End	test_data/sanger/ab1/OO230_2/1866_002_1_OO230_2_BR1EX8_F_M01.ab1	ab1	
OO230.2	OO230	ASSanger1	2014-01-27_15-55-13	Sanger	ABI Seq	Single-End	test_data/sanger/ab1/OO230_2/1866_003_1_OO230_2_BR1EX7_2_F_K01.ab1	ab1	
OO230.2	OO230	ASSanger1	2014-01-27_15-55-13	Sanger	ABI Seq	Single-End	test_data/sanger/ab1/OO230_2/1866_004_1_OO230_2_BR1EX7_1_F_I01.ab1	ab1	
OO230.2	OO230	ASSanger1	2014-01-27_15-55-13	Sanger	ABI Seq	Single-End	test_data/sanger/ab1/OO230_2/1866_005_1_OO230_2_BR1EX6_F_G01.ab1	ab1	
OO230.2	OO230	ASSanger1	2014-01-27_15-55-13	Sanger	ABI Seq	Single-End	test_data/sanger/ab1/OO230_2/1866_006_1_OO230_2_BR1EX5_F_E01.ab1	ab1	
OO230.2	OO230	ASSanger1	2014-01-27_15-55-13	Sanger	ABI Seq	Single-End	test_data/sanger/ab1/OO230_2/1866_007_1_OO230_2_BR1EX3_F_C01.ab1	ab1	
OO230.2	OO230	ASSanger1	2014-01-27_15-55-13	Sanger	ABI Seq	Single-End	test_data/sanger/ab1/OO230_2/1866_008_1_OO230_2_BR1EX2_F_A01.ab1	ab1	
OO230.2	OO230	ASSanger1	2014-01-27_15-55-01	Sanger	ABI Seq	Single-End	test_data/sanger/ab1/OO230_2/1866_009_1_OO230_2_BR1EX14_F_O05.ab1	ab1	
OO230.2	OO230	ASSanger1	2014-01-27_15-55-01	Sanger	ABI Seq	Single-End	test_data/sanger/ab1/OO230_2/1866_010_1_OO230_2_BR1EX13_F_M05.ab1	ab1	
OO230.2	OO230	ASSanger1	2014-01-27_15-55-01	Sanger	ABI Seq	Single-End	test_data/sanger/ab1/OO230_2/1866_011_1_OO230_2_BR1EX12_F_K05.ab1	ab1	

(b)

Patient_Id	Family_Id	Assay_Id	Lab_Analysis_Date	Platform	Seq_System	Read_Type	File_Path	File_Format	Barcode
p000001	F001	BRCA1_Illumina	2013-10-30_18-42-59	Illumina	HiSeq	Paired-End	test_data/illumina/SRR1611183_chr1_1_1M.fastq;test_data/illumina/SRR1611183_chr1_2_1M.fastq	fastq;fastq	
p000002	F001	BRCA1_Illumina	2013-10-30_18-42-59	Illumina	HiSeq	Paired-End	test_data/illumina/SRR1611183_chr1_1_5M.fastq;test_data/illumina/SRR1611183_chr1_2_5M.fastq	fastq;fastq	
p000003	F001	BRCA1_Illumina	2013-10-30_18-42-59	Illumina	HiSeq	Single-End	test_data/illumina/SRR1611183_chr1_1_1MS.fastq	fastq	
p000004	F001	BRCA1_Illumina	2013-10-30_18-42-59	Illumina	HiSeq	Single-End	test_data/illumina/SRR1611183_chr1_1_5MS.fastq	fastq	

(c)

Patient_Id	Family_Id	Assay_Id	Lab_Analysis_Date	Platform	Seq_System	Read_Type	File_Path	File_Format	Barcode
p000001	F001	AS454	2014-02-06_14-16-39	Roche	GS Junior	Single-End	test_data/454/fasta_qual/test1.fna;data/test_data/454/fasta_qual/test1.qual	fasta;qual	ACGAGTGCGT
p000002	F001	AS454	2014-02-06_14-16-39	Roche	GS Junior	Single-End	test_data/454/fasta_qual/test1.fna;data/test_data/454/fasta_qual/test1.qual	fasta;qual	ACGCTCGACA
p000003	F001	AS454	2014-02-06_14-16-39	Roche	GS Junior	Single-End	test_data/454/fasta_qual/test1.fna;data/test_data/454/fasta_qual/test1.qual	fasta;qual	AGACGCACTC
p000004	F001	AS454	2014-02-06_14-16-39	Roche	GS Junior	Single-End	test_data/454/fasta_qual/test1.fna;data/test_data/454/fasta_qual/test1.qual	fasta;qual	AGCACTGTAG
p000005	F001	AS454	2014-02-06_14-16-39	Roche	GS Junior	Single-End	test_data/454/fasta_qual/test1.fna;data/test_data/454/fasta_qual/test1.qual	fasta;qual	ATCAGACACG
p000006	F001	AS454	2014-02-06_14-16-39	Roche	GS Junior	Single-End	test_data/454/fasta_qual/test1.fna;data/test_data/454/fasta_qual/test1.qual	fasta;qual	ATATCGCGAG

(d)

Patient_Id	Family_Id	Assay_Id	Lab_Analysis_Date	Platform	Seq_System	Read_Type	File_Path	File_Format	Barcode
p000001	F001	ASPGM	2014-02-06_14-16-39	iontorrent	PGM	Single-End	test_data/iontorrent/sff/IOE5T0W01.sff	sff	ACGAGTGCGT
p000002	F001	ASPGM	2014-02-06_14-16-39	iontorrent	PGM	Single-End	test_data/iontorrent/sff/IOE5T0W01.sff	sff	ACGCTCGACA
p000003	F001	ASPGM	2014-02-06_14-16-39	iontorrent	PGM	Single-End	test_data/iontorrent/sff/IOE5T0W01.sff	sff	AGACGCACTC
p000004	F001	ASPGM	2014-02-06_14-16-39	iontorrent	PGM	Single-End	test_data/iontorrent/sff/IOE5T0W01.sff	sff	AGCACTGTAG
p000005	F001	ASPGM	2014-02-06_14-16-39	iontorrent	PGM	Single-End	test_data/iontorrent/sff/IOE5T0W01.sff	sff	ATCAGACACG
p000006	F001	ASPGM	2014-02-06_14-16-39	iontorrent	PGM	Single-End	test_data/iontorrent/sff/IOE5T0W01.sff	sff	ATATCGCGAG

(e)

Patient_Id	Family_Id	Assay_Id	Lab_Analysis_Date	Platform	Seq_System	Read_Type	File_Path	File_Format	Barcode
p000001	F01	ASA0001	2013-10-30_18-42-59	Illumina	HiSeq2000	Paired-End	data/test_data/illumina/NA12878/bam_files/p000001.bam	bam	
p000002	F01	ASA0001	2013-10-30_18-42-59	Illumina	HiSeq2000	Paired-End	data/test_data/illumina/NA12878/bam_files/p000002.bam	bam	

Figure 9: Shows different Target files for NGS data analysis. (a) Illumina analysis, (b) 454 analysis, (c) Ion torrent and (d) BAM files from any sequencing platform

2. Prepare MutAidOptions_NGS file

MutAid requires an input configuration file, which can be prepared once by customizing parameters as per the requirement, and the whole pipeline will be run without further user interaction.

(1) Set the **Global input parameters** as shown in Figure 10 below

```
################################################################################################################
#
###            Global input parameters
#
################################################################################################################

# Give the path to the fasta file of the genomic reference. The reference fasta should be obtained from UCSC genome browser
REFERENCEE_FASTA = ref_input/hg19_fa/chrAll.fa

# Give the gene annotation file of your genomic reference in GTF format. The reference GTF should be obtained from UCSC genome browser
REFERENCE_GTF_FILE = ref_input/hg19_gtf/hg19.gtf

### Give the number of processors that should be used in MutAid pipeline.
THREAD = 10

### Reference genome information from UCSC genome browser
Genome_Assembly = hg19
Refseq_Genome_Build = GRCh37


### dbSNP version from UCSC genome browser
dbSNP_Version = 142
```

Figure 10: Global input parameters of the MutAid pipeline.

(2) Set the **Output file and directory location** as shown in Figure 11 below

```
################################################################################################################
#
#                                    Output file and directory location
#
################################################################################################################

### Provide the output directory
Output_Dir = "test_output/454_output_dir"

### Provide the final output file name
Output_File = "test_output/454_output_file"
```

Figure 11: Set Output director and output file name and path

(3) Set the **Quality control and filtering** as shown in Figure 12 below

```
################################################################################################################
#
###            Step1: Quality control and filtering
#
################################################################################################################

### Minimum average base quality for trimming the low quality 5' and 3' end of reads
Minimum_Base_Quality = 20

### Minimum read length to discard the read for further analysis
Minimum_Read_Length = 50

### Maximum read length to discard the read for further analysis
Maximum_Read_Length = 1000

### Sanger data quality control and trimming parameters for TTUNER
Sanger_Trim_Window_Size = 10
Sanger_Trim_Base_Quality = 20
```

Figure 12: Set Quality control and filtering parameters

(4) Set the **Mapping to reference genome** parameters as shown in Figure 13 below

For Sanger data analysis default mapper is set as BWA mapper and Minimum

mapping quality threshold 20.

```
#########################################################################################################################
#
###             Step2: Mapping to reference genome
#
#########################################################################################################################

### Mapper name only one mapper at a time allowed. Example Mapper_Name = bwa
Mapper_Name = bwa

### Provide the Mapper_Parameters
Mapper_Parameters = ""
BWA_Sampe_Parameters = ""

### Filtering Mapped reads from BAM/SAM file
Minimum_Mapping_Quality = 20
```

Figure 13: Set Mapping parameters

(5) Set the **<u>Variant detection</u>** parameters as shown in Figure 14 below

```
#########################################################################################################################
#
###             Step1: Quality control and filtering
#
#########################################################################################################################

### Minimum average base quality for trimming the low quality 5' and 3' end of reads
Minimum_Base_Quality = 20

### Minimum read length to discard the read for further analysis
Minimum_Read_Length = 50

### Maximum read length to discard the read for further analysis
Maximum_Read_Length = 1000
```

Figure 14: Set Variant detection and filtering parameters

(6) Set the **<u>Variant functional annotation</u>** as shown in Figure 15 below

```
#########################################################################################################################
#
###             Step5: Variant functional annotation
#
#########################################################################################################################

## Various SNPs information file from UCSC Table browser
## Provide the full path to all these files.

UCSC_All_SNPs = ref_input/hg19_ucsc_dbxref/chrAll_snpAll.txt

UCSC_Common_SNPs = ref_input/hg19_ucsc_dbxref/chrAll_snpCommon.txt

UCSC_Flagged_SNPs = ref_input/hg19_ucsc_dbxref/chrAll_FlaggedSNPs.txt

UCSC_HapMap_SNPs = ref_input/hg19_ucsc_dbxref/chrAll_HapMapSNPs.txt

UCSC_Multi_SNPs = ref_input/hg19_ucsc_dbxref/chrAll_MultSNPs.txt

UCSC_COSMIC_SNPs = ref_input/hg19_ucsc_dbxref/chrAll_cosmic.txt

UCSC_GWAS_Catalog_SNPs = ref_input/hg19_ucsc_dbxref/chrAll_gwasCatalog.txt

UCSC_CpG_Islands_SNPs = ref_input/hg19_ucsc_dbxref/chrAll_cpgIslandExt.txt

UCSC_Coding_DbSnp = ref_input/hg19_ucsc_dbxref/chrAll_CodingDbSnp.txt

UCSC_OrthoPt3Pa2Rm2_SNPs = ref_input/hg19_ucsc_dbxref/chrAll_OrthoPt3Pa2Rm2.txt

UCSC_RefseqGene_Info = ref_input/hg19_ucsc_dbxref/chrAll_RefseqGene_Info.txt
```

Figure 15: Set the file path of these UCSC reference files

(7) Set the **<u>Third party software/tool executable path</u>** as shown in Figure 16 below

```
##################################################################################################################################
#
# Third party software/tool executables
# Provide the full path to the third party executables to run the MutAid pipeline.
#
#
##################################################################################################################################

### Get samtools to manipulate SAM and BAM files from http://samtools.sourceforge.net/
# Provide the full path of the executables relative to the <MutAid_1.0>
###### SAMTOOLS path
SAMTOOLS = executables/samtools-1.2/samtools
BCFTOOLS = executables/bcftools-1.2/bcftools


### Define the full path of the Mappers
BWA = executables/bwa-0.7.9a/bwa
TMAP = executables/TMAP/tmap
GSNAP = executables/gmap-2014-12-28/bin/gsnap
BOWTIE = executables/bowtie-1.1.1/bowtie
BOWTIE2 = executables/bowtie2-2.2.5/bowtie2

###### FASTQC executables
FASTQC = executables/FastQC/fastqc


###### AlienTrimmer for Adapter and Primer and Homopolymer trimming
AlienTrimmer = executables/AlienTrimmer_0.4.0/src/AlienTrimmer.jar

###### varient callers path
GATK = executables/GenomeAnalysisTK.jar
GATK_BUNDLE = ref_input/GATK/bundle

#FreeBayes = executables/freebayes/bin/freebayes
Varscan2 = executables/VarScan.v2.3.7.jar


#### PICARD JAR file from http://picard.sourceforge.net/index.shtml
###### 3 PICARD jar files are required for MutAid pipeline
PICARD = executables/picard-tools-1.130/picard.jar
AddOrReplaceReadGroups = executables/picard-tools-1.115/picard-tools-1.115/AddOrReplaceReadGroups.jar
BuildBamIndex = executables/picard-tools-1.115/picard-tools-1.115/BuildBamIndex.jar
MarkDuplicates = executables/picard-tools-1.115/picard-tools-1.115/MarkDuplicates.jar
MergeSamFiles = executables/picard-tools-1.115/picard-tools-1.115/MergeSamFiles.jar
SortSam = executables/picard-tools-1.115/picard-tools-1.115/SortSam.jar
ReorderSam = executables/picard-tools-1.115/picard-tools-1.115/ReorderSam.jar
CreateSequenceDictionary = executables/picard-tools-1.115/picard-tools-1.115/CreateSequenceDictionary.jar


### Get intersectBed executables from a collection of useful utilities called bedtools-
### from http://code.google.com/p/bedtools/
###### 3 bedtools
intersectBed = executables/bedtools2/bin/intersectBed
bedtools = executables/bedtools2/bin/bedtools
bamToBed = executables/bedtools2/bin/bamToBed
```

Figure 16: Specifications of full paths of external software/tools used in MutAid.

Step2: Run MutAid whole pipeline:

After preparing the Target File and MutAidOptions file have been prepared and customized then MutAid pipeline can be run with following command line

MutAid_v1.0/mutaid –option_file MutAidOptions_NGS

Step2: Run MutAid pipeline step-by-step:

Alternatively user can run MutAid pipeline form NGS data analysis in a step-by-step manner. The main advantage of this feature is, user can optimize the parameters for each step independently without spending time to finish whole

pipeline. Once all parameters have been optimized then providing the MutAidOptions_NGS file can run whole pipeline.

Since NGS data are enormous in size and in coverage thus we have facilitated MutAid whole pipeline with many start and stop points and thus user can run 1) Only quality control and filtering step, 2) Only Mapping step and 3) Only variant calling and 4) Only Variant effect prediction, variant annotation and write final variant summary table output.

Run MutAid only for Quality control and filtering

./mutaid –option_file MutAidOptions_NGS --qc

Run MutAid only for Mapping

./mutaid –option_file MutAidOptions_NGS --map

Run MutAid only variant detection

./mutaid –option_file MutAidOptions_NGS --variant_call

Run MutAid for only writing output

With this command MutAid 1) predicts genomic effects like codon change, amino acid change and genomic feature assignment 2) Functional and clinical annotation of all resulting variants and 3) write final output variant summary table.

./mutaid –option_file MutAidOptions_NGS --write_output

7. MutAid outputs description

After running MutAid, the results of the MutAid pipeline can be found in the output directory (as specified in the MutAid Options file). Results are provided in the following format.

```
<OUTPUT_FILE>
<OUTPUT_DIR>
        |
        - <fastq_files_after_qc>
                |
                - <patient1_1.fq;patient1_2.fq>
                - <patient2_1.fq;patient2_2.fq>
                - <patient3_1.fq;patient3_2.fq>
                - .
                - .
                - .
                - <patientN_1.fq;patientN_2.fq>

        - <QC_report>
                |
                - <before_qc_patient1_1.fq_fastqc_qc_report.html>
                - <after_qc_patient1_1.fq_fastqc_qc_report.html>
                - <before_qc_patient1_2.fq_fastqc_qc_report.html>
                - <after_qc_patient1_2.fq_fastqc_qc_report.html>
                - .
                - .
                - .       |
                - .
                - <before_qc_patientN_1.fq_fastqc_qc_report.html>
                - <after_qc_patientN_1.fq_fastqc_qc_report.html>
                - <before_qc_patientN_2.fq_fastqc_qc_report.html>
                - <after_qc_patientN_2.fq_fastqc_qc_report.html>

        - < bam_files>
                |
                - <patient1.bam>
                - <patient2.bam>
                - <patient3.bam>
                - .
                - .
                - .
                - <patientN.bam>
        - <variant_files>
                |
                - <patient1.vcf>
                - <patient2.vcf>
                - <patient3.vcf>
```

- .
- .
- .
- <patientN.vcf>

<fastq_files_after_qc>:

This output folder contains high quality FASTQ file for each patient with Sanger quality encoding. If reads are in paired-end then there will be two files for each patient.

<QC_report>:

This output folder contains Quality control and trimming report in html format generated by FASTQC tool. There are two files for each FASTQ files 1) before quality control and 2) after quality control.

<bam_files>:

This output folder contains resulting BAM file along with BAM index for each patient/sample. These BAM files have been generated after applying all mapping parameters and post-mapping filtering criteria.

<variant_files>:

This output folder contains resulting variants (SNV, Insertion, and Deletion) in Variant Call Format (VCF) for each patient/sample.

8. Contact Information

PD Dr. Andreas Weinhäusel
andreas.weinhaeusel@ait.ac.at
Dr. Albert Kriegner
albert.kriegner@platomics.com
Ram Vinay Pandey
ramvinay.pandey@gmail.com
