# Supplementary material for: MutAid: Sanger and NGS Based Integrated Pipeline for Mutation Identification, Validation and Annotation in Human Molecular Genetics
Source: PLoS One. 2016 Feb 3;11(2):e0147697. doi: 10.1371/journal.pone.0147697 (PMC4739551; doi:10.1371/journal.pone.0147697)
Supplement: S2 Appendix — (PDF) [file pone.0147697.s007.pdf]

MutAid: Sanger and NGS based integrated pipeline for mutation identification, validation and annotation in human molecular genetics.

Ram Vinay Pandey[1,2*], Stephan Pabinger[1], Albert Kriegner[1] and Andreas Weinhäusel[1]

[1]AIT Austrian Institute of Technology, Health and Environment Department, Molecular Diagnostics, Vienna, Austria.

[2]Institut für Populationsgenetik, Vetmeduni Vienna, Veterinärplatz 1, A-1210 Vienna, Austria

*Corresponding author

E-mail: ramvinay.pandey@gmail.com

Fisher's exact test method

Fisher's exact test generally operates on contingency tables [1]. It can be applied to allele counts of biallelic SNV between selected SNV and the remaining SNVs in a given patient/sample. Fisher's exact test can be used to rank the potential candidate SNV based on significant p-value.

Here, we demonstrate how Fisher's exact test is performed in MutAid for each Single Nucleotide Variant (SNV) within a Patient/Sample data. First we construct a 2x2 contingency table for each SNV and then run the Fisher's exact test using R [1-2] and finally we perform the multiple testing corrections by using the False Discovery Rate (FDR) correction.

We run Fisher's exact test with two-tailed hypothesis at 95% confidence interval.

Here we have described the process of Fisher's exact test.

Example:

A Patient has 10 SNV as shown in table 1 below:

Table 1: shows 10 SNVs in a given Patient/Sample

The Ref_allele_count is the number of high quality mapped reads support reference allele and Variant_allele_count is the number of high quality mapped reads support variant allele.

SNV	Ref_allele_count	Variant_allele_count
SNV1	106	67
SNV2	491	136

SNV3	558	47
SNV4	69	61
SNV5	119	26
SNV6	321	121
SNV7	255	59
SNV8	153	42
SNV9	112	85
SNV10	155	88

Fisher's exact test for SNV1

Step1: Construction of contingency table

We construct a 2x2 contingency table for SNV1. As shown in Table 2 below, the green color highlighted row is the current SNV (SNV1) for which Fisher's exact test is performed.

Table 2: shows the 10 SNV in table, where SNV1 is selected (highlighted in green color) for Fisher's exact test.

SNV	Ref_allele_count	Variant_allele_count
SNV1	106	67
SNV2	491	136
SNV3	558	47

SNV4	69	61
SNV5	119	26
SNV6	321	121
SNV7	255	59
SNV8	153	42
SNV9	112	85
SNV10	155	88
**Total Sum**	**2233**	**665**

The 2x2 contingency table for SNV1 is constructed as explained below.

In contingency table (Table 3), Row 1 is the count of Reference allele and variant allele count in Column 1 and column 2 respectively.

The second row is the "**Total Sum**" from Table 2.

Row 2 Column 1 total sum = Ref_allele_count of [SNV2 + SNV3 + SNV4 SNV5 SNV6 SNV7 + SNV8 + SNV9 + SNV10]

Row 2 Column 2 total sum = Variant_allele_count *of [SNV2 + SNV3 + SNV4 SNV5 SNV6 SNV7 + SNV8 + SNV9 + SNV10]*

**From Sum SNV1 is excluded.*

Table 3: shows the 2x2 contingency table for SNV1

	Column 1	Column 2
Row 1	106	67
Row 2	2233	665

Step2:

Run Fisher's exact test for contingency table in R [2] as given R commands below:

```
x<-matrix(c(106,2233,67,665), nrow=2)
fisher.test(x, alternative = "two.sided", conf.level = 0.95, simulate.p.value=T, B=100000)
```

Step3:

After Fisher’s exact test we performs multiple testing correction (FDR) in R [2-3] with given R command.

First read all p-value calculated in step 2 with following R command

```
all_pvalue=read.table("fisher_exact_test_output_p_value", header=F,sep="\t", na.strings="na")
```

Run FDR multiple testing correction with following R command

```
p.adjust(all_pvalue, method="fdr", n=length(all_pvalue))"
```

The "BH" also called "fdr" is the method of Benjamini, Hochberg, and Yekutieli control the false discovery rate, the expected proportion of false discoveries amongst the rejected hypotheses. The false discovery rate (FDR) is a less stringent condition than the family-wise error rate, so these methods are more powerful than the others [3].

Fisher’s exact test for SNV2

Step1: Construction of contingency table

We construct a 2x2 contingency table for SNV2. As shown in Table 4 below, the green color highlighted row is the current SNV (SNV2) for which Fisher’s exact test is performed.

Table 4: shows the 10 SNV in table, where SNV2 is selected (highlighted in green color) for Fisher’s exact test.

SNV	Ref_allele_count	Variant_allele_count
SNV1	106	67
SNV2	491	136
SNV3	558	47
SNV4	69	61
SNV5	119	26

SNV6	321	121
SNV7	255	59
SNV8	153	42
SNV9	112	85
SNV10	155	88
**Total Sum**	**1848**	**596**

The 2x2 contingency table for SNV2 is constructed as explained below.

In this table, Row 1 is the count of reference allele and variant allele count in Column 1 and column 2 respectively.

The second row is the "**Total Sum**" from Table 4 as shown above.

Row 2 Column 1 total sum = Ref_allele_count of [SNV1 + SNV3 + SNV4 SNV5 SNV6 SNV7 + SNV8 + SNV9 + SNV10]

Row 2 Column 2 total sum = Variant_allele_count *of [SNV1 + SNV3 + SNV4 SNV5 SNV6 SNV7 + SNV8 + SNV9 + SNV10]*

**From Sum SNV2 is excluded.*

Table 5: shows the 2x2 contingency table for SNV2

	Column 1	Column 2
Row 1	491	136
Row 2	1848	596

Step2:

Run Fisher's exact test for contingency table in R [2] as given R commands below:

```
x<-matrix(c(491,1848,136,596), nrow=2)
fisher.test(x, alternative = "two.sided", conf.level = 0.95, simulate.p.value=T, B=100000)
```

Step3:

After Fisher's exact test we performs multiple testing correction (FDR) in R [2-3] with given R command.

First read all p-value calculated in step 2 with following R command

```
all_pvalue=read.table("fisher_exact_test_output_p_value", header=F,sep="\t", na.strings="na")
```

Run FDR multiple testing correction with following R command

```
p.adjust(all_pvalue, method="fdr", n=length(all_pvalue))
```

The "BH" also called "fdr" is the method of Benjamini, Hochberg, and Yekutieli [3] control the false discovery rate, the expected proportion of false discoveries amongst the rejected hypotheses. The false discovery rate (FDR) is a less stringent condition than the family-wise error rate, so these methods are more powerful than the others [3].

References

1. Fisher RA. On the interpretation of χ2from contingency tables, and the calculation of P. J. R. Stat. Soc. 1922; 85:87-94.
2. R statistics [https://cran.r-project.org/].
3. Benjamini, Y., and Yekutieli, D. (2001). The control of the false discovery rate in multiple testing under dependency. Ann. Statist. Volume 29, Number 4 (2001), 1165-1188.
