# Supplementary figures and images for: MutAid: Sanger and NGS Based Integrated Pipeline for Mutation Identification, Validation and Annotation in Human Molecular Genetics
Source: PLoS One. 2016 Feb 3;11(2):e0147697. doi: 10.1371/journal.pone.0147697 (PMC4739551; doi:10.1371/journal.pone.0147697)

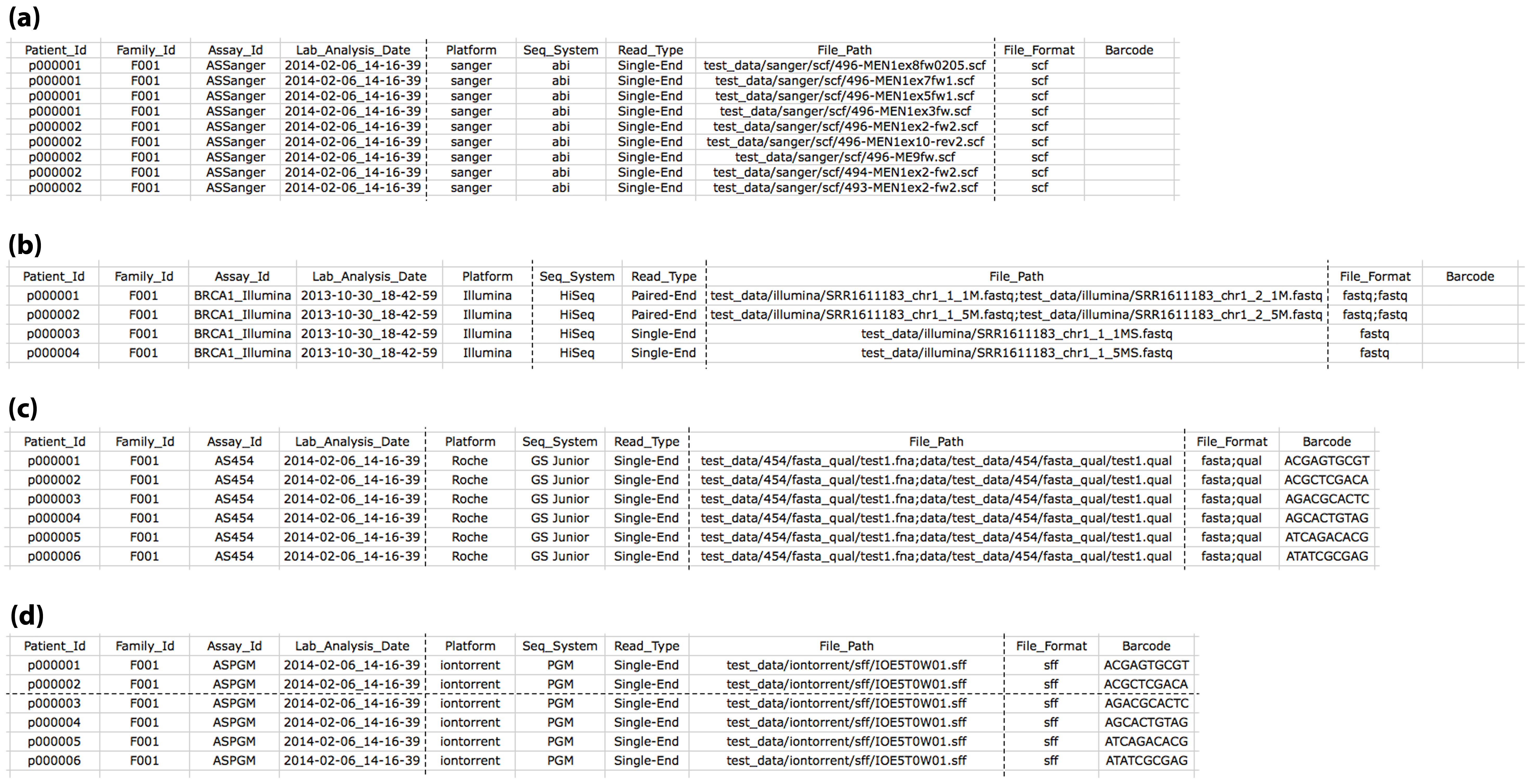

Supplement: S1 Fig — (TIF) [file pone.0147697.s008.tif]
